# Facile Synthesis of 5-Arylidene Thiohydantoin by Sequential Sulfonylation/Desulfination Reaction

**DOI:** 10.3390/ijms140612484

**Published:** 2013-06-13

**Authors:** Jintao Han, Hongbo Dong, Zhihong Xu, Jianping Lei, Mingan Wang

**Affiliations:** 1 Department of Applied Chemistry, China Agricultural University, Beijing 100193, China; E-Mails: hanjint321@163.com (J.H.); bloodwhenseeme@163.com (H.D.); x_u_78@sina.com (Z.X.); leijianping2006@163.com (J.L.); 2 College of Agriculture, Yangtze University, Jingzhou 434025, Hubei, China

**Keywords:** 5-arylidene thiohydantoin, sulfonylation, desulfination, arylsulfonyl chloride

## Abstract

The sequential sulfonylation/desulfination reactions of 5-benzylthiohydantoin with excess arylsulfonyl chlorides in the presence of triethylamine have been developed to afford a wide range of 5-arylidene thiohydantoin derivatives in moderate to excellent yields. A plausible sulfonylation/desulfination mechanism was proposed. The bioassay showed that these compounds exhibit certain fungicidal activities with the 71.9% inhibition rate of **2K** against *B. cinerea*, and 57.6% inhibition rate of **2m** against *A. solani*, respectively.

## 1. Introduction

**Scheme 1:**
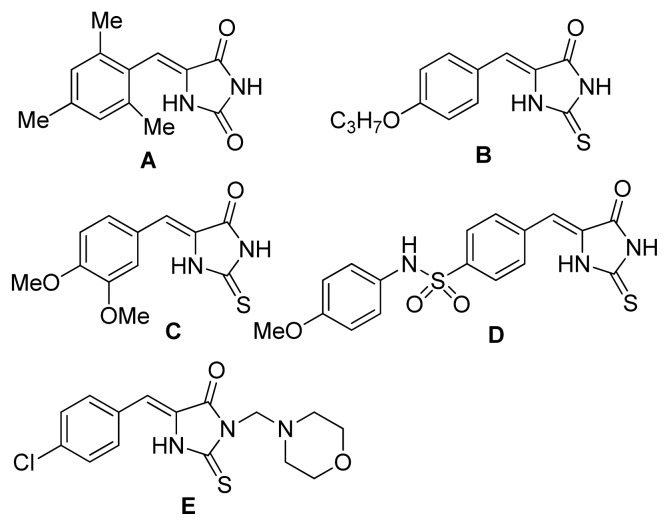
The biological active 5-arylidene hydantoin and thiohydantoin compounds.

5-Arylidene hydantoins and thiohydantoins are key structural motifs of numerous natural and unnatural products that display a wide range of biological activity. Among them, hydantoin **A** has exhibited comparable anticonvulsant activity to phenytoin [1], **B** showed antimycobacterial activity against *Mycobacterium tuberculosis* with IC_50_ of 4.5 μg/mL [2], and **C** and **D** exhibited fungicidal activity with 95%–100% inhibition against *Erysiphe graminis*, *Uromyceis appendiculatus*, and *Botrytis cinerea* [3], **E** had been investigated as antitumor agent (Scheme 1). In addition, these 5-arylidene derivatives are very useful building blocks for the synthesis of various heterocycles such as 5-arylidene derivatives of imidazoline-4-one, imidazothiazine, diazinone, and diazepinone [4–8]. In 5-arylidene hydantoins and thiohydantoins, the arylidene carbon-carbon double bond at 5-position are as important as hydantoin or thiohydantoin moiety to the biological effect [1–3]. Therefore, there has been great demand for highly efficient synthetic methods for the introduction of arylidene carbon-carbon double bond at the 5-position of the hydantoin or thiohydantoin ring [1,2]. So far, several typical synthetic strategies of 5-arylidene derivatives of hydantoin and thiohydantoin have been reported, including a traditional base-catalyzed aldol condensation of hydantoin and thiohydantoin with substituted benzaldehydes [1,3,5,6,9], improved aldol condensation such as one-pot multicomponent reaction of substituted benzaldehydes, glycine and potassium thiocyanate [10], microwave-assisted condensation of thiohydantoin/hydantoin with substituted benzaldehyde Schiff bases [11], Wittig-Horner Reaction [12–14], and Mn-catalyzed reactions of terminal alkynes with isocyanates [15]. Herein, we report a new protocol *via* sequential sulfonylation/desulfination to construct the 5-arylidene thiohydantoin.

## 2. Results and Discussion

**Figure 1:**
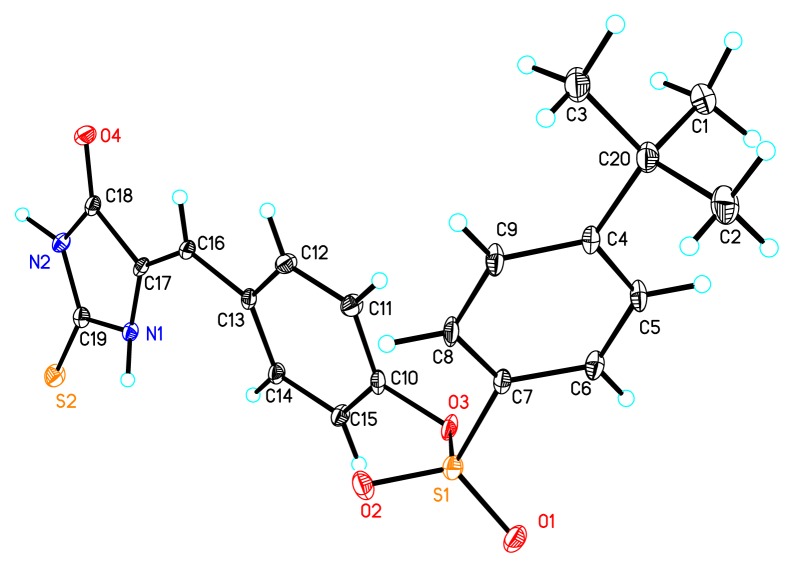
X-ray structure of compound **2k**.

In our laboratory, we needed to prepare 5-aryl derivatives of hydantoin and thiohydantoin as the key intermediate to develop the novel inhibitor of Adenylosuccinate Synthetase (AdSS) [16–18], which plays a key role in the two-step conversion of IMP to AMP in the *de novo* pathway of purine biosynthesis [19–21]. In order to prepare 5-aryl derivatives of thiohydantoin, 5-(4-hydroxylbenzyl)-thiohydantoin was treated with phenylsulfonyl chloride in acetone at 30 °C in the presence of one equivalent of triethyl amine (entry 1, Table 1). The target product **3a** was obtained in 84% yield. In addition, unexpectedly, a new product **2a** was isolated in 15% yield. We established the structure of the new product **2a** to be that from sequential sulfonylation/desulfination at the 5-position of thiohydantoin, using NMR spectroscopy and X-ray crystallography of the homologous **2k** obtained from the reaction of **1** with the *p*-tert-butyl-phenylsulfonyl chloride **4k** (Figure 1, see more in supplementary Figures S1 and S2, Tables S1–S3), and natural occurring compound 5-(4-hydroxy phenylidene)-2-thiohydantoin [22]. The reaction provided the trisubstituted exocyclic alkylidene as a single *Z* isomer [23]. Next, we examined the sulfonylation/desulfination reaction in detail. Table 1 presents the results of screening for appropriate reaction conditions for the model reaction between **1** and phenylsulfonyl chloride **4a**. When the amount of phenylsulfonyl chloride was increased to 1.2, 1.5, 2 and 3 equation, the corresponding product **2a** was obtained in 29%, 69% and 81%, 80% yields, respectively (entries 2–5, Table 1). Therefore, the best quantity of phenylsulfonyl chloride was 2 equivalents for this reaction. When the reaction times were shortened, the yields decreased from 81% to 38% (entry 4,6,7,8, Table 1). As the temperature was decreased to 0 °C, the yield decreased to 53%, while as the temperature was increased to 60 °C, the yield was kept 81% (entry 9,10, Table 1). When the reaction solvents and the bases were canged, the yields decreased greatly (entry 11–15, Table 1). If we added 3–5 equivalents of Et_3_N, the yields also decreased (entry 16–18, Table 1). The following reactions were then performed for 8h in 1:2:2 ratios for 5-(4-hydroxylbenzyl)-thiohydantoin, phenylsulfonyl chloride and Et_3_N in acetone solution at 30 °C.

Under the optimized reaction conditions, we examined a range of arylsulfonyl chlorides in the sequential sulfonylation/desulfination of 5-(4-hydroxybenzyl)-2-thioxoimidazolidin-4-one (**1**). As shown in Table 2, a variety of arylsulfonyl chlorides underwent the sulfonylation/desulfination at the 5-position of thiohydantoin, providing the anticipated 5-arylidene thiohydantoin **2** in moderate to excellent yields (entries 1–14, Table 2). Arylsulfonyl chlorides featuring phenyl groups with either electron-withdrawing or -donating substituents worked well as substrates under the given conditions.

Considering that the sulfonyl is a strong electron-withdrawing group, we assumed that 5-aryl-2-thioxoimidazolidin-4-ones having another electron-withdrawing group on the benzene ring will also work. As indicated in Table 3, 5-benzylthiohydantoins were synthesized and treated with phenylsulfonyl chloride under the optimized conditions. We were pleased to observe that the anticipated products 5-phenylidene-thiohydantoin were obtained in 38%–86% yields (entries 1–9, Table 3). In a sharp contrast with these substrates, 5-(4-aminobenzyl)-thiohydantoin having a strong electron-donating NH_2_ group on the benzene ring did not carry out the reaction (entries 10, Table 3). In addition, the reaction of 5-aryl derivatives of hydantoin **3a** had been explored to give **2a** in 91% yield under the given conditions with one equivalent molar of phenylsulfonyl chloride (**4a**). Interestingly, 5-benzylthiohydantoins undertook a similar reaction using phenylsulfonyl chloride and *N*,*N*-diisopropylethylamine (DIPEA) without changing the other reaction conditions (entries 11–13, Table 3). It should be stated that all of the prepared compounds **2** and **6** exhibit similar *Z* selectivity based on the ^1^H NMR analysis of significant methine proton chemical shifts (Figure S1) and comparison with the data in the references [1–6].

**Figure 2:**
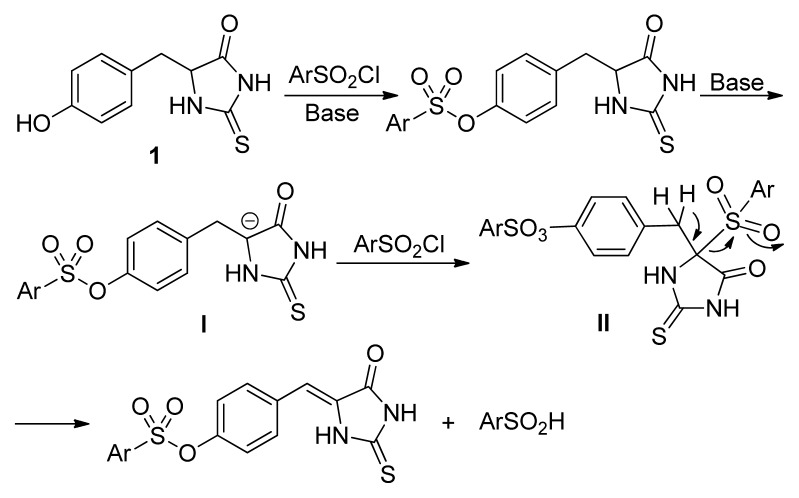
The proposed reaction mechanism of 5-arylidene thiohydantoin.

Based on the above results, we proposed a sequential proton leaving-phenylsulfone formation-phenylsulfinic acid elimination mechanism for the reaction (Figure 2). After the sulfonylation of the starting material **1**, the acidity of proton at the 5-postion of thiohydantoin was enhanced. Consequent deprotonation in the presence of base afforded the carbon anion, which was captured by phenylsulfonyl chloride to give intermediate **II**. Then, the sulfone intermediate **II** lost one molar of phenylsulfinic acid to give 5-arylidene thiohydantoin. This mechanism was very similar to *Ei* elimination observed by Jenks in the sulfone chemistry, in which sulfone groups act simultaneously as base and leaving group [25,26], an enone and conjugated olefin preparation via phenylsulfinic acid elimination of β-sulfone developed by Trost and Procopiou groups [27–29], and thermal decomposition pathway for polysulfones found by Wudl [30].

The data in Table 4 showed that these compounds exhibit certain inhibition rates against *Botrytis cinerea*, *Alternaria solani*, *Fusarium oxysporum* and *Dothiorella gregaria* at the concentration of 100 μg/mL. In general, the fungicidal activities against *B. cinerea* and *A. solani* were better than that of against *F. oxysporum* and *D. gregaria*. The inhibition rate of **2k** against *B. cinerea* was 71.9%, and the inhibition rate of **2m** against *A. solani* was 57.6%, respectively. They are much better than that of carbendazim (the positive control) against *B. cinerea* and *A. solani*.

## 3. Experimental Section

### 3.1. General Information

All reactions were performed under an air atmosphere with magnetic stirring. Unless otherwise stated, all reagents were purchased from commercial suppliers and used without further purification. Organic solutions were concentrated under reduced pressure using a rotary evaporator or oil pump. Flash column chromatography was performed using Qingdao Haiyang flash silica gel (200–300 mesh). Meting points were measured on a Yanagimoto apparatus and uncorrected. Infrared spectra were recorded using a Shimadzu IR-435 instrument with KBr plates. ^1^H and ^13^C NMR spectra were obtained on Bruker DPX 300 spectrometer with DMSO-*d*_6_ as a solvent and TMS as an internal standard. Elemental analysis was performed on a Vario EL instrument (Elementar Vario Micro Cube, Hanau, Germany). X-ray crystallographic data were collected using a Bruker SMART CCD-based diffractometer operated at 293 K. Crystallographic for compound **2K** have been deposited at the Cambridge Crystallographic data center, CCDC No. 831811. Copies of this information may be obtained free of charge from the Director, CCDC, 12 Union Road, Cambridge, CB2, 1EZ, UK.

### 3.2. Synthesis

#### 3.2.1. The Synthesis of 5-(4-Hydroxybenzyl)-Thiohydantoin (**1**)

A 1:3 mixture of tyrosine (5.4 g, 30 mmol) and thiourea (6.9 g, 90 mmol) was placed in a flask and heated under stirring. Control the oil bath temperature to 180–190 °C, about 5 min later the homogenous liquid started to fume. After 15 min, the reaction was completed as monitored by TLC. The flask was allowed to cool down and 20 mL water was added while the flask was still warm. The solution was reheated to dissolve all the solids and allowed to cool to room temperature, then placed in a refrigerator for 4 h. The crystal of compound **1** was removed by vacuum filtration, and the mother liquid was extracted with ethyl acetate and further purified by flash column chromatography. Finally, 5.3 g yellow product was obtained. Yield: 83%. m.p. 208–209 °C (Literature: 211 °C [32]).^1^H NMR (DMSO-d_6_, 300 MHz) δ: 11.39 (s, 1H), 10.04 (s, 1H), 9.25 (s, 1H), 6.94 (d, *J* = 8.7 Hz, 2H), 6.63 (d, *J* = 8.7 Hz, 2H), 4.45 (dd, *J* = 4.8, 3.9 Hz, 1H), 2.85 (d, *J* = 4.8 Hz, 2H).

#### 3.2.2. General Procedure for the Synthesis of Compounds **2** and **6**

To a stirred solution of 5-(4-hydroxybenzyl)-2-thiohydantoin or 5-substitutedbenzyl-2-thiohydantoin (2 mmol) and Et_3_N or DIPEA(4 mmol) in acetone (15 mL), substituted phenylsulfonyl chloride (2–6 mmol) was added dropwise at 0 °C, then the temperature was allowed to 0, 30, 60 °C. The reaction was monitored by TLC. After 2–8 h, stop the reaction, and filtered. The products were purified by column chromatography on silica gel (petrolem ether-EtOAc, 1.5:1) to give the product **2** and **6**. The ^1^H and ^13^C NMR spectra for new compounds could be found in supplementary Figure S1.

5-(Phenylsulfonyloxy) phenylidene thiohydantoin **2a**, yellow solid, m.p. 199–200 °C. ^1^H NMR (DMSO-d_6_, 300 MHz) δ: 12.42 (s, 1H), 12.17 (s, 1H), 7.91–7.04 (m, 9H), 6.45 (s, 1H); ^13^C NMR (DMSO-d_6_, 75 MHz) δ: 179.62, 165.78, 149.08, 135.26, 134.44, 131.83, 129.96, 128.47, 128.33, 122.41, 109.68; IR (KBr) ν: 3470, 3329, 1780, 1725, 1649 cm^−1^. Anal calcd for C_16_H_12_N_2_O_4_S_2_: C 53.32, H 3.36, N 7.77; Found: C 53.26, H 3.19, N 7.72.

5-(Phenylsulfonyloxy) benzyl thiohydantoin **3a**, white solid**,** m.p. 158–160 °C. ^1^H NMR (DMSO-d_6_, 300 MHz) δ: 11.51 (s, 1H), 10.05 (s, 1H), 7.84–7.64 (m, 5H), 7.17–7.14 (m, 2H), 6.93–6.88 (m, 2H), 4.54 (dd, *J* = 4.4, 5.3 Hz, 1H), 2.96 (d, *J* = 5.3 Hz, 1H). IR (KBr) *ν*: 3123, 2869, 1746, 1544, 1502, 1377, 1177, 1154, 1090, 823 cm^−1^.

5-(4-Flurophenylsulfonyloxy) phenylidene thiohydantoin **2b**, yellow solid, m.p. 227–228 °C, ^1^H NMR (DMSO-d_6_, 300 MHz) δ: 12.41 (s, 1H), 12.15 (s, 1H), 7.99–7.06 (m, 8H), 6.45 (s, 1H); ^13^C NMR (DMSO-d_6_, 75 MHz) δ:179.69, 165.86, 165.84 (*J*_CF_ = 254.9 Hz), 149.01, 131.98, 131.92 (*J*_CF_ = 13.0 Hz), 131.74, 130.69, 128.61, 122.53, 117.41 (*J*_CF_ = 23.1 Hz), 109.69; IR (KBr) ν: 3465, 3262, 1780, 1730, 1653cm^−1^. Anal calcd for C_16_H_11_FN_2_O_4_S_2_: C 50.79, H 2.93, N 7.40; Found: C 50.79, H 2.76, N 7.28.

5-(4-Chlorophenylsulfonyloxy) phenylidene thiohydantoin **2c**, yellow solid, m.p.206–208 °C, ^1^H NMR (DMSO-d_6_, 300 MHz) δ: 12.43 (s, 1H), 12.18 (s, 1H), 7.93–7.08 (m, 8H), 6.45 (s, 1H); ^13^C NMR (DMSO-d_6_, 75 MHz) δ: 179.65, 165.76, 148.93, 140.34, 133.19, 131.98, 131.91, 130.30, 130.19, 128.55, 122.47, 109.62; IR (KBr) ν: 3448, 3179, 1776, 1733, 1648 cm^−1^; Anal calcd for C_16_H_11_ClN_2_O_4_S_2_: C 48.67, H 7.09, N 2.81; Found: C 48.58, H 7.06, N 2.53.

5-(4-Chlorophenylsulfonyloxy) benzyl thiohydantoin **3c**, white solid, m.p. 168–170 °C. ^1^H NMR (DMSO-d_6_, 300 MHz) δ: 11.52 (s, 1H), 10.04 (s, 1H), 7.79–7.72 (m, 4H), 7.20–7.16 (m, 2H), 6.96–6.92 (m, 2H), 4.55 (dd, *J* = 4.4, 5.3 Hz, 1H), 2.97 (d, *J* =5.0 Hz, 1H). IR (KBr) *ν*: 3212, 2881, 1743, 1548, 1504, 1477, 1379, 1154, 1094, 825 cm^−1^.

5-(4-Methoxyphenylsulfonyloxy) phenylidene thiohydantoin **2d**, yellow solid, m.p. 208–210 °C, ^1^H NMR (DMSO-d_6_, 300 MHz) δ: 12.41 (s, 1H), 12.16 (s, 1H), 7.82–7.03 (m, 8H), 6.45 (s, 1H), 3.87 (s, 3H); ^13^C NMR (DMSO-d_6_, 75 MHz) δ: 179.67, 165.87, 164.32, 149.30, 131.87, 131.73, 130.86, 128.50, 125.66, 115.23, 115.23, 109.87, 56.16; IR (KBr) ν: 3420, 3257, 1768, 1652 cm^−1^; Anal calcd for C_17_H_14_N_2_O_5_S_2_: C 52.30, H 3.61, N 7.17; Found: C 52.16, H 3.40, N 7.18.

5-(4-Methoxyphenylsulfonyloxy) benzyl thiohydantoin **3d**, white solid, m.p. 181–183 °C, ^1^H NMR (DMSO-d_6_, 300 MHz) δ: 11.50 (s, 1H), 10.04 (s, 1H), 7.70–7.65 (m, 2H), 7.17–7.13 (m, 4H), 6.91–6.88 (m, 2H), 4.54 (dd, *J* = 4.4, 5.3 Hz, 1H), 3.87 (s, 3H), 2.96 (d, 1H, *J* = 5.0 Hz). IR (KBr) *ν*: 3150, 3024, 2928, 1742, 1592, 1502, 1372, 1268, 1151, 1092, 862, 844 cm^−1^.

5-(4-Methylphenylsulfonyloxy) phenylidene thiohydantoin **2e**, yellow solid, m.p. 184–185 °C, ^1^H NMR (DMSO-d_6_, 300 MHz) δ: 12.42 (s, 1H), 12.17 (s, 1H), 7.78–7.04 (m, 8H), 6.45 (s, 1H), 2.43 (s, 3H); ^13^C NMR (DMSO-d_6_, 75 MHz) δ: 179.62, 165.78, 149.17, 146.06, 131.83, 131.73, 131.57, 130.42, 128.45, 128.36, 122.41, 109.74, 21.31; IR (KBr) ν: 3472, 3387, 1785, 1730, 1649 cm^−1^; Anal calcd for C_17_H_14_N_2_O_4_S_2_: C 54.53, H 3.77, N 7.48; Found: C 54.46, H 3.87, N 7.52.

5-(4-Nitrophenylsulfonyloxy) phenylidene thiohydantoin **2f**, yellow solid, m.p. 258–260 °C, ^1^H NMR (DMSO-d_6_, 300 MHz) δ: 12.43 (s, 1H), 12.18 (s, 1H), 8.48–7.11 (m, 8H), 6.45 (s,1H); ^13^C NMR (DMSO-d_6_, 75 MHz) δ:179.68, 165.77, 151.25, 148.76, 139.63, 132.22, 132.01, 130.19, 128.66, 125.19, 122.46, 109.53; IR (KBr) ν: 3468, 3284, 1775, 1736, 1651 cm^−1^; Anal calcd for C_16_H_11_N_3_O_6_S_2_: C 47.70, H 2.73, N 10.36; Found: C 47.70, H 2.59, N 10.23.

5-(4-Bromophenylsulfonyloxy) phenylidene thiohydantoin **2g**, yellow solid, m.p. 230–232 °C, ^1^H NMR (DMSO-d_6_, 300 MHz) δ: 12.43 (s, 1H), 12.19 (s, 1H), 7.93–7.90 (d, *J* = 8.8 Hz, 2H), 7.83–7.80 (d, *J* = 8.8 Hz, 2H), 7.77–7.74 (d, *J* = 8.7 Hz, 2H), 7.10–7.07 (d, *J* = 8.7 Hz, 2H), 6.46(s, 1H); ^13^C NMR (DMSO-d_6_, 75 MHz) δ: 179.68, 165.82, 148.96, 133.63, 133.18, 132.03, 131.96, 130.33, 129.56, 128.58, 122.52, 109.68; IR (KBr) ν: 3414, 3246, 3181, 1734, 1650 cm^−1^; Anal calcd for C_16_H_11_BrN_2_O_4_S_2_: C 43.74, H 2.52, N 6.38; Found: C 43.76, H 2.57, N 6.40.

5-(2-Methylphenylsulfonyloxy) phenylidene thiohydantoin **2h**, yellow solid, m.p. 160–162 °C, ^1^H NMR (DMSO-d_6_, 300 MHz) δ: 12.42 (s, 1H), 12.16 (s, 1H), 7.79–7.69 (m, 5H), 7.46–7.02 (m, 3H), 6.46 (s, 1H); 2.72 (s, 3H); ^13^C NMR (DMSO-d_6_, 75 MHz) δ: 179.63, 165.80, 149.05, 138.34, 135.24, 133.23, 131.89, 131.79, 130.44, 130.24, 128.47, 127.04, 122.16, 109.69, 19.99; IR (KBr) ν: 3382, 3155, 1760, 1606 cm^−1^; Anal calcd for C_17_H_14_N_2_O_4_S_2_: C 54.53, H 3.77, N 7.48; Found: C 54.48, H 3.68, N 7.38.

5-(3-Trifluromethylphenylsulfonyloxy) phenylidene thiohydantoin **2i**, yellow solid, m.p. 196–198 °C, ^1^H NMR (DMSO-d_6_, 300 MHz) δ: 12.44 (s, 1H), 12.18 (s, 1H), 8.28–8.16 (m, 3H), 7.98–7.95 (d, *J* = 7.9 Hz, 1H), 7.78–7.75 (d, *J* = 8.7 Hz, 2H), 7.73–7.11 (d, *J* = 8.7 Hz, 2H), 6.46 (s, 1H); ^13^C NMR (DMSO-d_6_, 75 MHz) δ: 179.68, 165.80, 148.79, 135.58, 132.58, 132.15, 131.96, 131.77, 128.61, 124.97, 122.52, 109.56; Anal calcd for C_17_H_11_F_3_N_2_O_4_S_2_: C 43.74, H 2.52, N 6.38; Found: C 43.76, H 2.57, N 6.40.

5-(4-Iodophenylsulfonyloxy) phenylidene thiohydantoin **2j**, yellow solid, m.p. 210–212 °C, ^1^H NMR (DMSO-d_6_, 300 MHz) δ: 12.43 (s, 1H), 12.18 (s,1H), 8.10–8.07 (d, *J* = 8.9 Hz, 2H), 7.77–7.74 (d, *J* = 8.8 Hz, 2H), 7.64–7.61 (d, *J* = 8.7 Hz, 2H), 7.10–7.07 (d, *J* = 8.7 Hz, 2H), 6.46 (s, 1H); ^13^C NMR (DMSO-d_6_, 75 MHz) δ: 179.65, 165.80, 148.96, 138.95, 133.97, 131.97, 131.93, 129.97, 128.55, 122.47, 109.68, 104.41; IR (KBr) ν: Anal calcd forC_16_H_11_IN_2_O_4_S_2_: C 39.52, H 2.28, N 5.76; Found: C 39.73, H 2.49, N 5.76.

5-(4-*tert*-Butylphenylsulfonyloxy) phenylidene thiohydantoin **2k**, yellow solid, m.p. 210–212 °C, ^1^H NMR (DMSO-d_6_, 300 MHz) δ: 12.42 (s, 1H), 12.17 (s,1H), 7.85–7.82 (d, *J* = 8.8 Hz, 2H), 7.77–7.70 (m, 4H), 7.10–7.07 (d, *J* = 8.9 Hz, 2H), 6.46 (s, 1H), 1.32 (s, 9H); ^13^C NMR (DMSO-d_6_, 75 MHz) δ: 179.62, 165.80, 158.53, 149.16, 131.87, 131.82, 131.73, 128.46, 128.21, 126.85, 122.35, 109.76, 35.29, 30.78; IR (KBr) ν: Anal calcd for C_20_H_20_N_2_O_4_S_2_: C 57.67, H 4.84, N 6.73; Found: C 57.56, H 4.92, N 6.73.

5-(4-*tert*-Butylphenylsulfonyloxy) benzyl thiohydantoin **3k**, white solid**,** m.p. 161–163 °C, ^1^H NMR (DMSO-d_6_, 300 MHz) δ: 11.50 (s, 1H), 10.04 (s, 1H), 7.74–7.67 (m, 4H), 7.19–7.15 (m, 2H), 6.95–6.90 (m, 2H), 4.55 (dd, *J* = 4.4, 5.3 Hz, 1H), 2.96 (d, *J* = 5.3 Hz, 1H), 1.32 (s, 9H). IR (KBr) *ν*: 3167, 3042, 2962, 1740, 1596, 1547, 1403, 1373, 1184, 1089, 889, 825 cm^−1^.

5-(4-Acetylaminophenylsulfonyloxy) phenylidene thiohydantoin **2l**, yellow solid, m.p. 262–264 °C, ^1^H NMR (DMSO-d_6_, 300 MHz) δ: 12.41 (s, 1H), 12.17 (s, 1H), 10.50 (s, 1H), 7.86–7.72 (m, 6H), 7.04–7.01 (d, *J* = 8.8 Hz, 2H), 6.44 (s, 1H), 2.11 (3H, s); ^13^C NMR (DMSO-d_6_, 75 MHz) δ: 179.61, 169.49, 165.79, 149.20, 145.15, 131.81, 131.69, 129.86, 128.42, 127.30, 122.50, 118.86, 109.77, 24.34; IR (KBr) ν: Anal calcd for C_18_H_15_N_3_O_5_S_2_: C 51.79, H 3.62, N 10.07; Found: C 51.80, H 3.76, N 9.97.

5-(2,5-Dichlorophenylsulfonyloxy) phenylidene thiohydantoin **2m**, yellow solid, m.p. 226–228 °C, ^1^H NMR (DMSO-d_6_, 300 MHz) δ: 12.44 (s, 1H), 12.19 (s, 1H), 7.95–7.92 (m, 3H), 7.79–7.75 (d, *J* = 8.9 Hz, 2H), 7.18–7.15 (d, *J* = 8.9 Hz, 2H), 6.46 (s, 1H); ^13^C NMR (DMSO-d_6_, 75 MHz) δ: 179.70, 165.79, 148.68, 136.61, 134.51, 133.93, 132.31, 132.41, 130.89, 128.73, 122.05, 109.52; IR (KBr) ν: Anal calcd for C_16_H_10_Cl_2_N_2_O_4_S_2_: C 44.76, H 2.35, N 6.53; Found: C 44.82, H 2.57, N 6.42.

5-(3,4-Dichlorophenylsulfonyloxy) phenylidene thiohydantoin **2n**, yellow solid, m.p. 220–222°C, ^1^H NMR (DMSO-d_6_, 300 MHz) δ: 12.42 (s, 1H), 12.15 (s, 1H), 8.19 (s, 1H), 7.98–7.95 (d, *J* = 8.5 Hz, 1H), 7.85–7.77 (m, 3H), 7.17–7.14 (d, *J* = 8.9 Hz, 2H), 6.47 (s, 1H); ^13^C NMR (DMSO-d_6_, 75 MHz) δ: 179.68, 165.80, 148.81, 138.68, 134.51, 133.14, 132.29, 131.98, 129.98, 128.67, 128.48, 122.54, 109.58; IR (KBr) ν: Anal calcd for C_16_H_10_Cl_2_N_2_O_4_S_2_: C 44.76, H 2.35, N 6.53; Found: C 45.01, H 2.60, N 6.52.

### 3.3. Bioassay of Fungicidal Activity

Fungicidal activities of compounds **2** against *B. cinerea*, *A.solani*, *F. oxysporurm*, and *D. gregaria* were evaluated using the mycelium growth rate test [33]. The culture media with known concentration of the test compounds were obtained by mixing the solution of **2** in acetone with potato dextrose agar (PDA), on which fungus cakes were placed. The blank test was made using acetone and carbendazin was used as positive control. The culture was incubated at 25 ± 0.5 °C. Three replicates were performed. After the mycelium in the blank grew completely, the diameter of the mycelium was measured and the inhibition rate calculated according to the formula in reference [33]. In which I is the inhibition rate, P_0_ is the average diameter of the mycelium in the blank, and P_1_ is the average diameter of the mycelium in the presence of the test samples. Mean measurements were calculated from the three replicates.

## 4. Conclusions

In conclusion, we have investigated the sequential sulfonylation/desulfination reaction of 5-(4-hydroxylbenzyl)-thiohydantoin with excess arylsulfonyl chloride in the presence of triethylamine. These reactions are operationally simple and proceed smoothly under very mild reaction conditions, providing a broad range of 5-arylidene thiohydantoin derivatives in moderate to excellent yields. The further applications of the reaction in the synthesis of biologically relevant molecules are ongoing in our laboratory. The bioassay showed that these compounds exhibit certain fungicidal activities with the 71.9% inhibition rate of **2K** against *B. cinerea*, and 57.6% inhibition rate of **2m** against *A. solani*, respectively.

## Supplementary Information



## Figures and Tables

**Table 1:** The reaction of **1** and phenylsulfonyl chloride (**4a**).

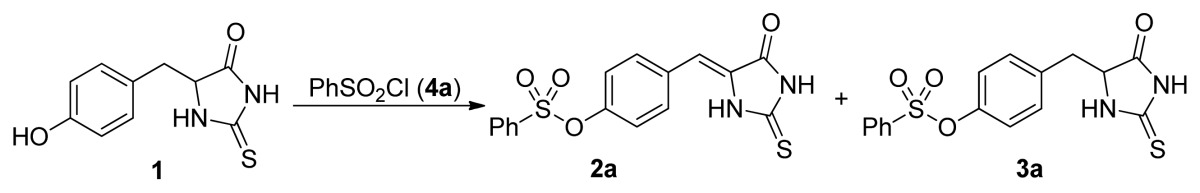							

Entry	Ratio (1:4a)	Temp (°C)	Time (h)	Solvent	Base	2a Yields (%) a	3a yield (%) a
1	1:1	30	8	acetone	Et_3_N	15	84 [24]
2	1:1.2	30	8	acetone	Et_3_N	29	58 [24]
3	1:1.5	30	8	acetone	Et_3_N	69	23 [24]
4	1:2	30	8	acetone	Et_3_N	81	<5 [24]
5	1:3	30	8	acetone	Et_3_N	80	- b
6	1:2	30	6	acetone	Et_3_N	76	- b
7	1:2	30	4	acetone	Et_3_N	49	- b
8	1:2	30	2	acetone	Et_3_N	38	- b
9	1:2	0	8	acetone	Et_3_N	53	- b
10	1:2	60	8	acetone	Et_3_N	81	- b
11	1:2	30	8	CH_2_Cl_2_	Et_3_N	41	- b
12	1:2	30	8	CHCl_3_	Et_3_N	63	- b
13	1:2	30	8	THF	Et_3_N	66	- b
14	1:2	30	8	acetone	Pyridine	34	- b
15	1:2	30	8	acetone	Na_2_CO_3_	28	- b
16 c	1:2	30	8	acetone	Et_3_N	63	- b
17 d	1:2	30	8	acetone	Et_3_N	64	- b
18 e	1:2	30	8	acetone	Et_3_N	65	- b

a Isolated yield;

b The side-product **3a** hadn’t been isolated;

c–e The 3–5 equivalents of Et_3_N were used, respectively.

**Table 2:** The reaction of **1** and arylsulfonyl chloride (**4**).

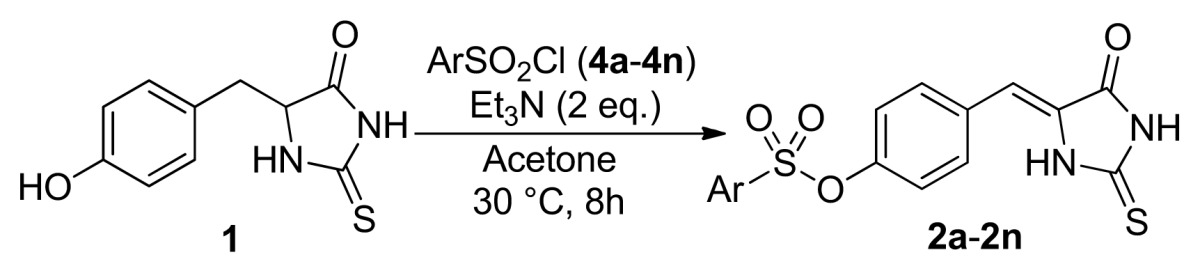			
Entry	Ar	Product	Yield (%)
1	Ph (**4a**)	**2a**	81
2	4-FC_6_H_4_ (**4b**)	**2b**	78
3	4-ClC_6_H_4_ (**4c**)	**2c**	77
4	4-MeOC_6_H_4_ (**4d**)	**2d**	40
5	4-MeC_6_H_4_ (**4e**)	**2e**	46
6	4-NO_2_C_6_H_4_ (**4f**)	**2f**	84
7	4-BrC_6_H_4_ (**4g**)	**2g**	49
8	2-MeC_6_H_4_ (**4h**)	**2h**	42
9	3-CF_3_C_6_H_4_ (**4i**)	**2i**	80
10	4-IC_6_H_4_ (**4j**)	**2j**	49
11	4-*t*-ButC_6_H_4_ (**4k**)	**2k**	80
12	4-AcNHC_6_H_4_ (**4l**)	**2l**	48
13	2,5-Cl_2_C_6_H_4_ (**4m**)	**2m**	52
14	3,4-Cl_2_C_6_H_4_ (**4n**)	**2n**	54

**Table 3:** The reaction of analogues **5** and arylsulfonyl chloride (**4a**).

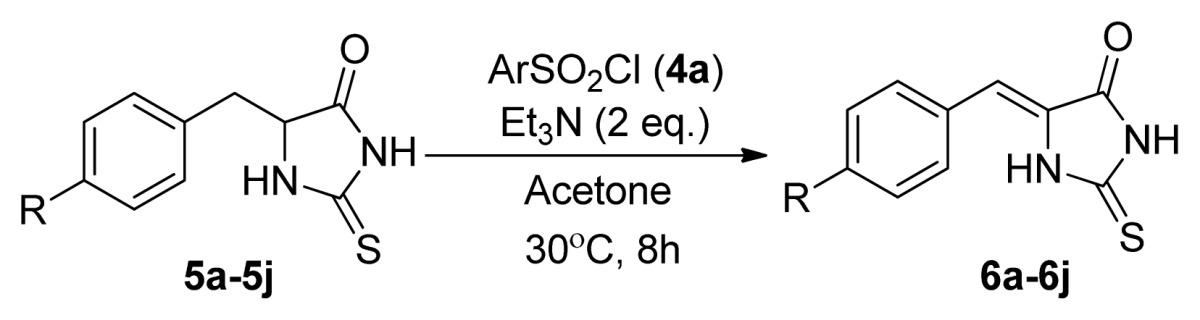			
Entry	R	Product a	Yield (%)
1	H (**5a**)	**6a**	38
2	4-F (**5b**)	**6b**	77
3	4-Cl (**5c**)	**6c**	74
4	4-Br (**5d**)	**6d**	65
5	4-Me (**5e**)	**6e**	48
6	4-NO_2_ (**5f**)	**6f**	76
7	4-MeO (**5g**)	**6g**	49
8	4-CF_3_ (**5h**)	**6h**	86
9	2,4-Cl_2_ (**5i**)	**6i**	75
10	4-NH_2_ (**5j**)	**6j**	0
11	H (**5a**)	**6a**	52
12	4-NO_2_ (**5f**)	**6f**	84
13	4-CF_3_ (**5h**)	**6h**	88

a The products were identified by ^1^H NMR and MS data [2,31].

**Table 4:** The fungicidal activities (inhibition rate, %) of compounds **2** against several plant fungi.

Compd.	*Botrytis cinerea*	*Alternaria solani*	*Fusarium oxysporum*	*Dothiorella gregaria*
**2a**	27.6	55.4	37.2	−5.3
**2b**	12.6	27.7	23.4	−6.4
**2c**	21.8	37.3	18.1	−5.3
**2d**	3.4	22.9	8.5	−3.2
**2e**	17.2	48.2	29.4	1.1
**2f**	10.6	24.7	5.6	−7.0
**2g**	12.5	35.1	30.9	29.7
**2h**	10.4	26.8	20.3	11.2
**2i**	29.3	33.2	0	38.4
**2j**	19.4	15.9	0	0
**2k**	71.9	48.9	22.9	17.6
**2l**	0	34.1	0	13.9
**2m**	27.8	57.6	31.9	36.8
**2n**	21.6	19.3	9.0	0
Carbendazim	8.4	10.9	100	97.6
