# Supplementary material for: Facile Synthesis of 5-Arylidene Thiohydantoin by Sequential Sulfonylation/Desulfination Reaction
Source: Int J Mol Sci. 2013 Jun 13;14(6):12484–95. doi: 10.3390/ijms140612484 (PMC3709795; doi:10.3390/ijms140612484)
Supplement: Supplementary file 1 [file ijms-14-12484-s001.pdf]

Supplementary Information

Figure S1. ^{1}H and ^{13}C NMR spectra of compounds **2a**–**2n**, **3a**–**3k**.

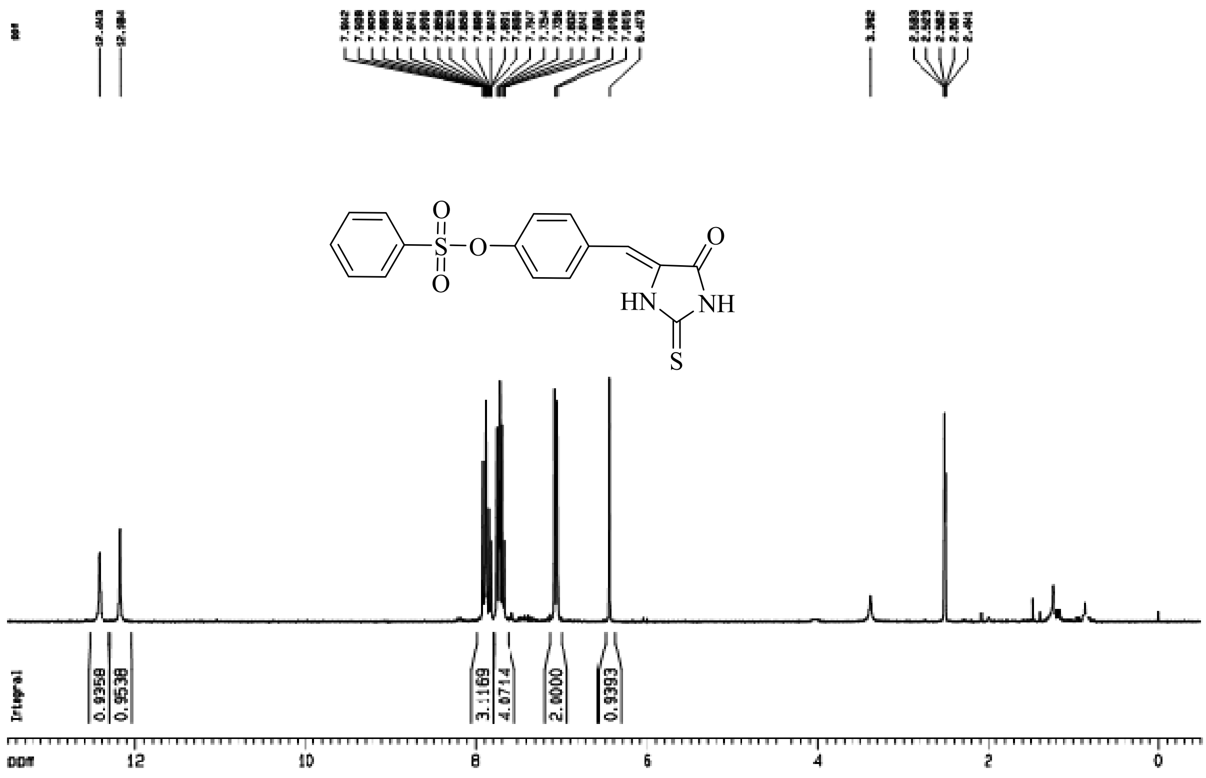

^{1}H NMR of **2a**

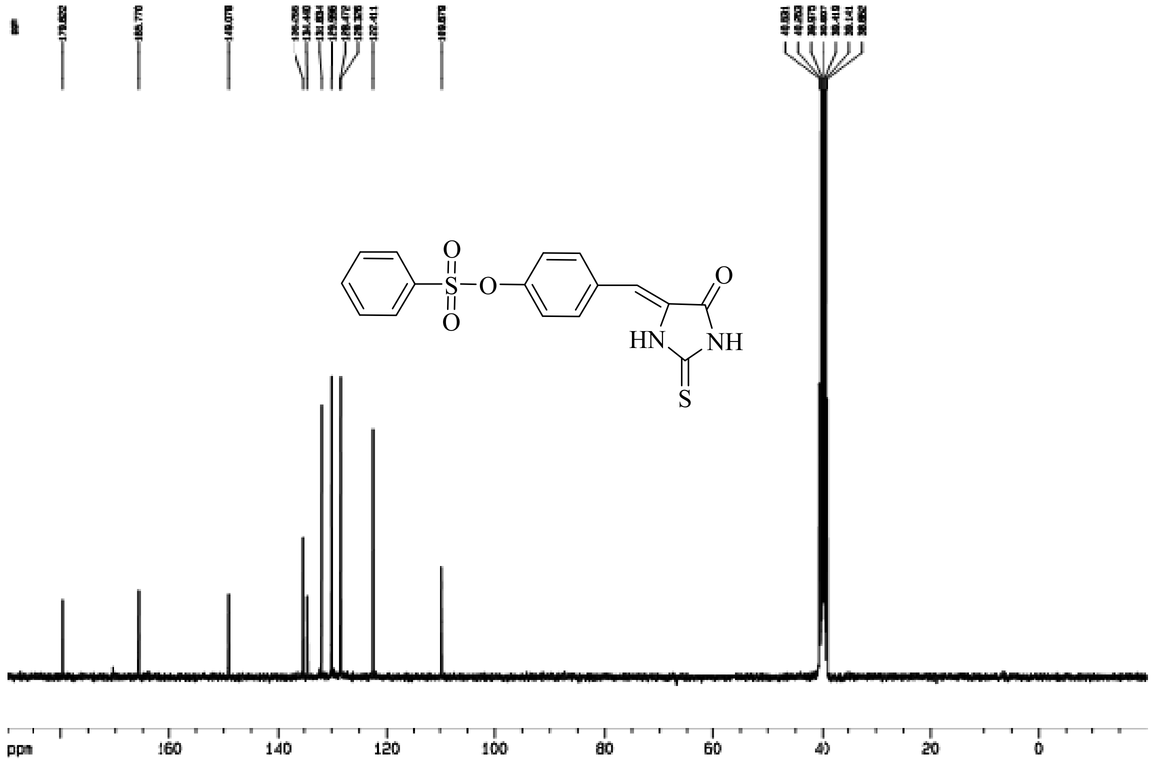

^{13}C NMR of **2a**

Figure S1. *Cont.*

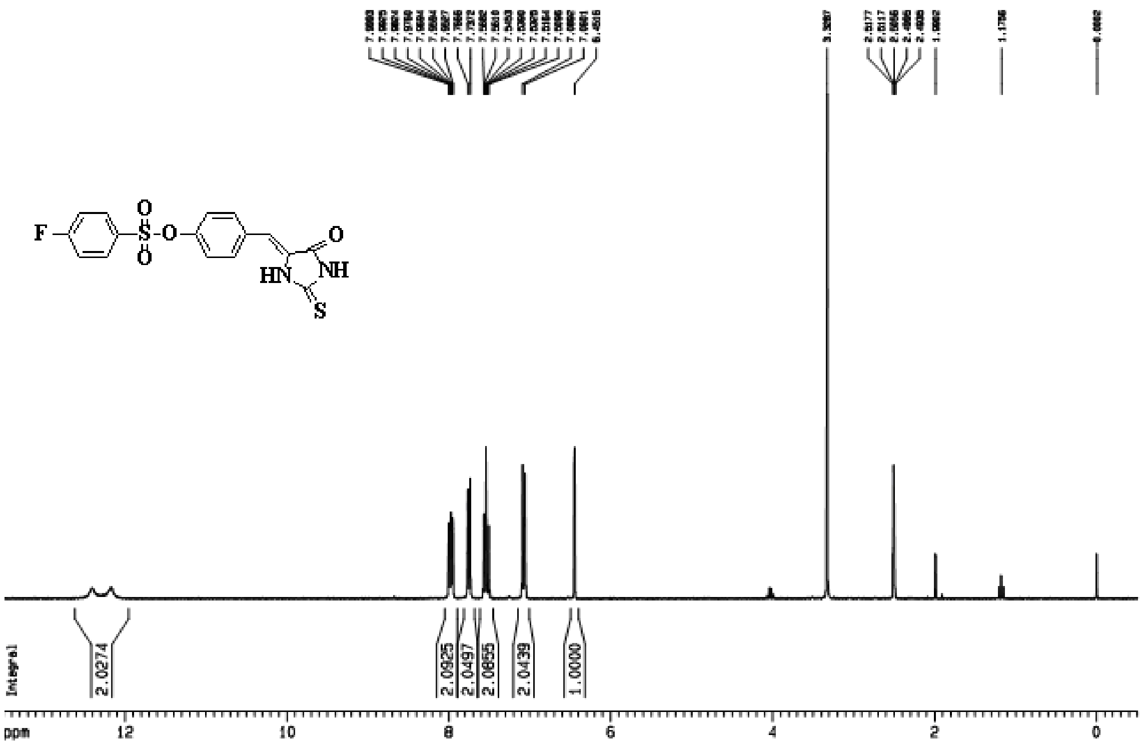

^{1}H NMR of **2b**

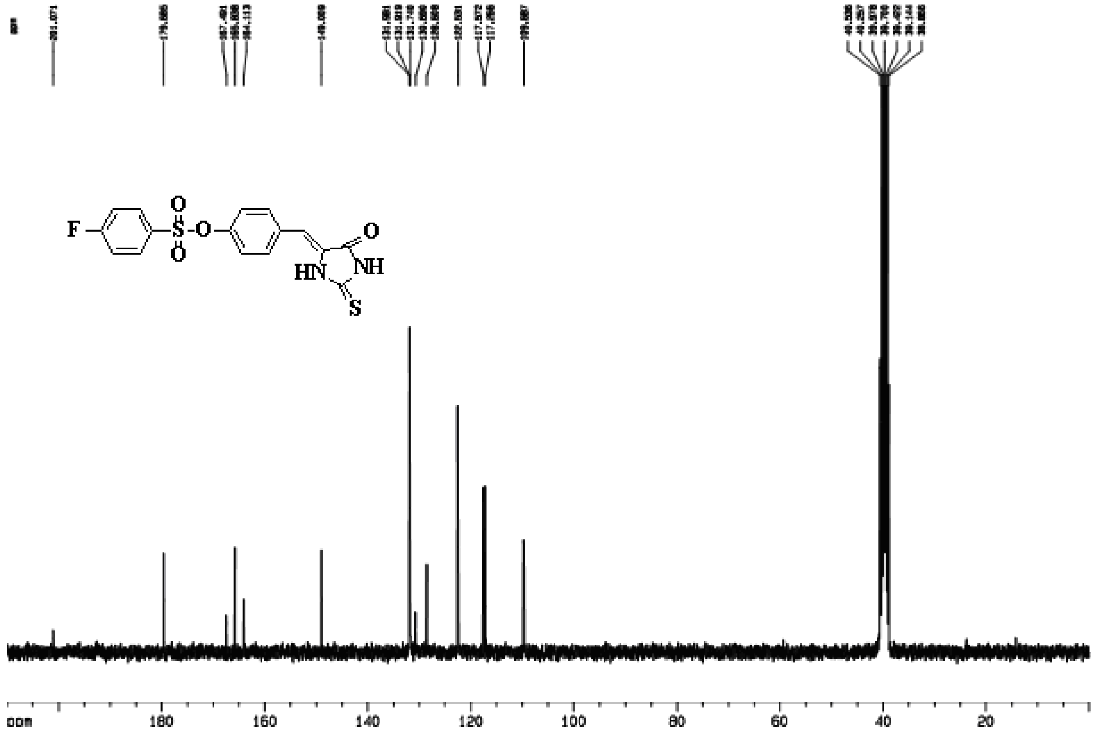

^{13}C NMR of **2b**

Figure S1. *Cont.*

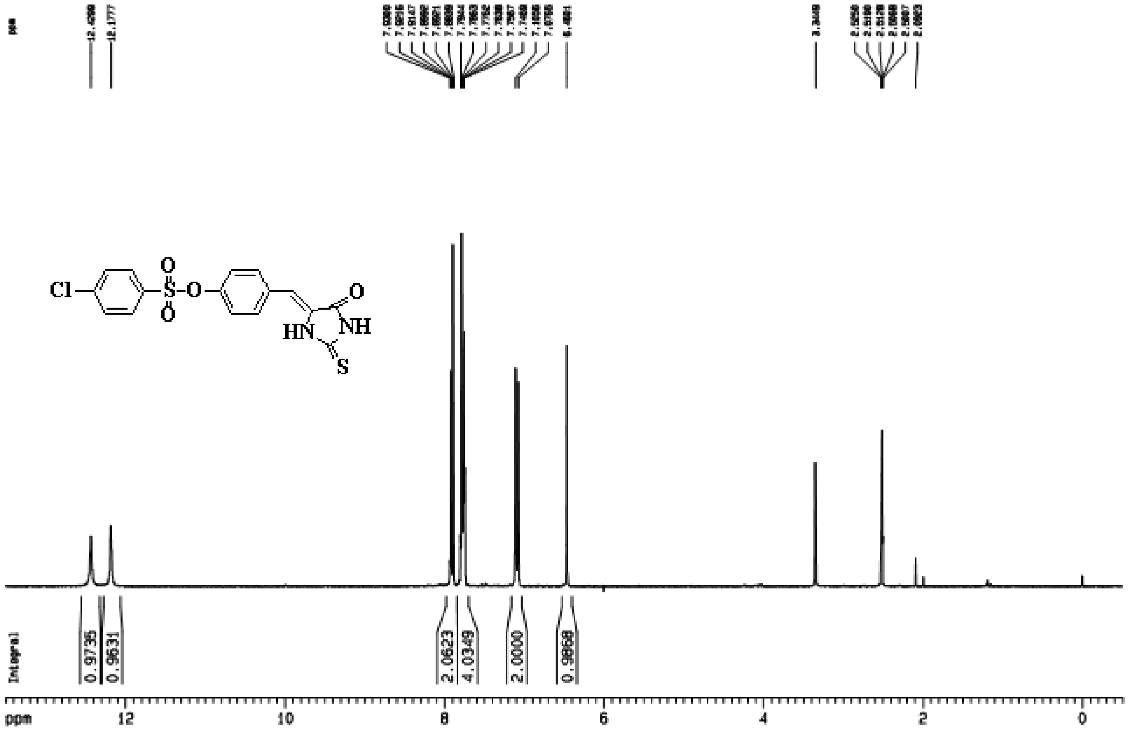

^{1}H NMR of **2c**

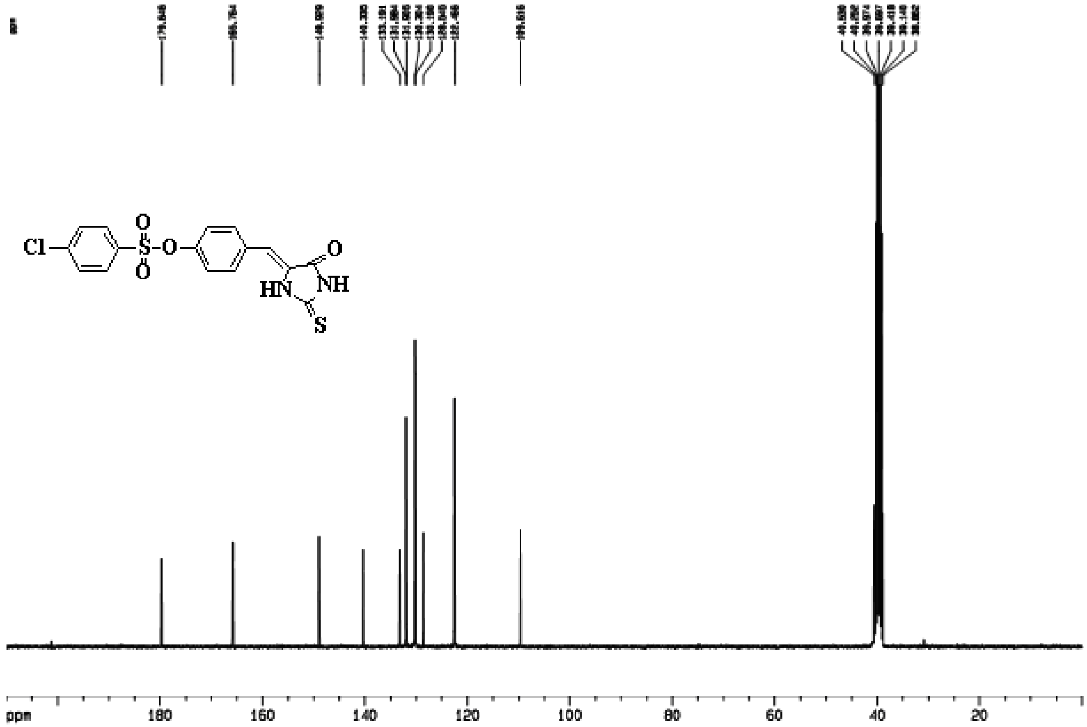

^{13}C NMR of **2c**

Figure S1. *Cont.*

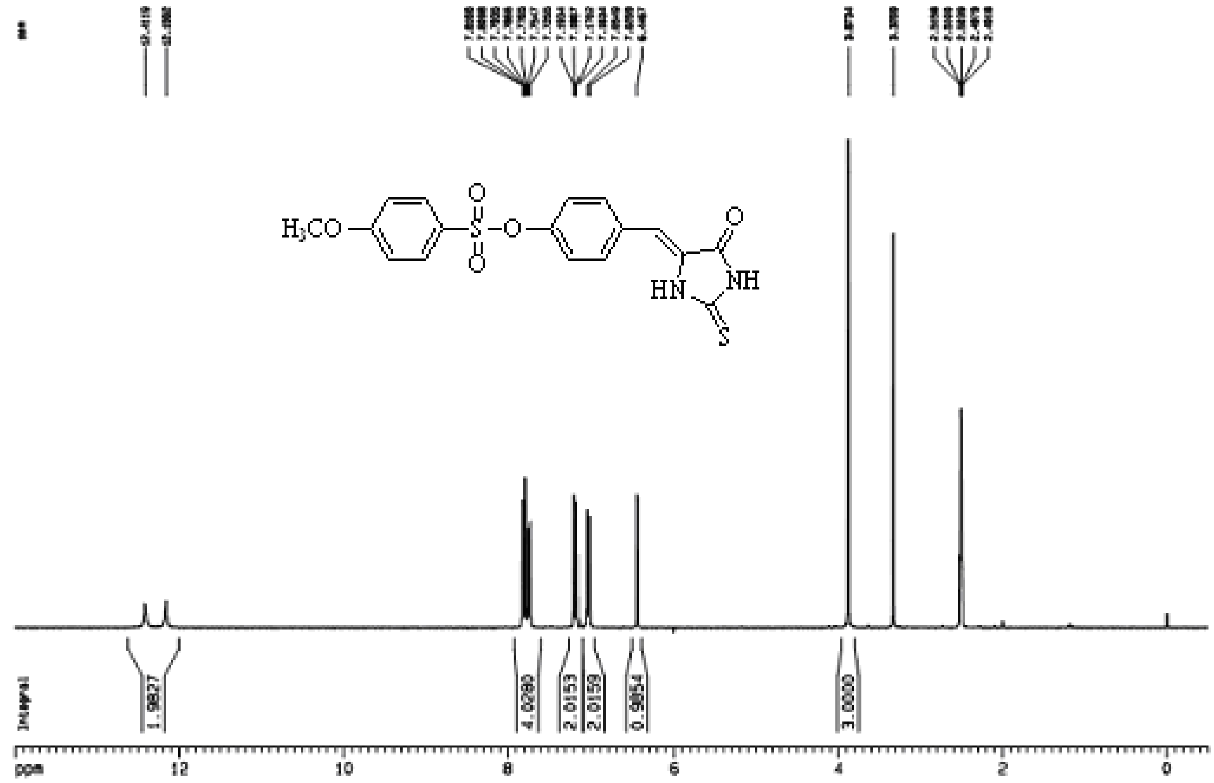

^{1}H NMR of **2d**

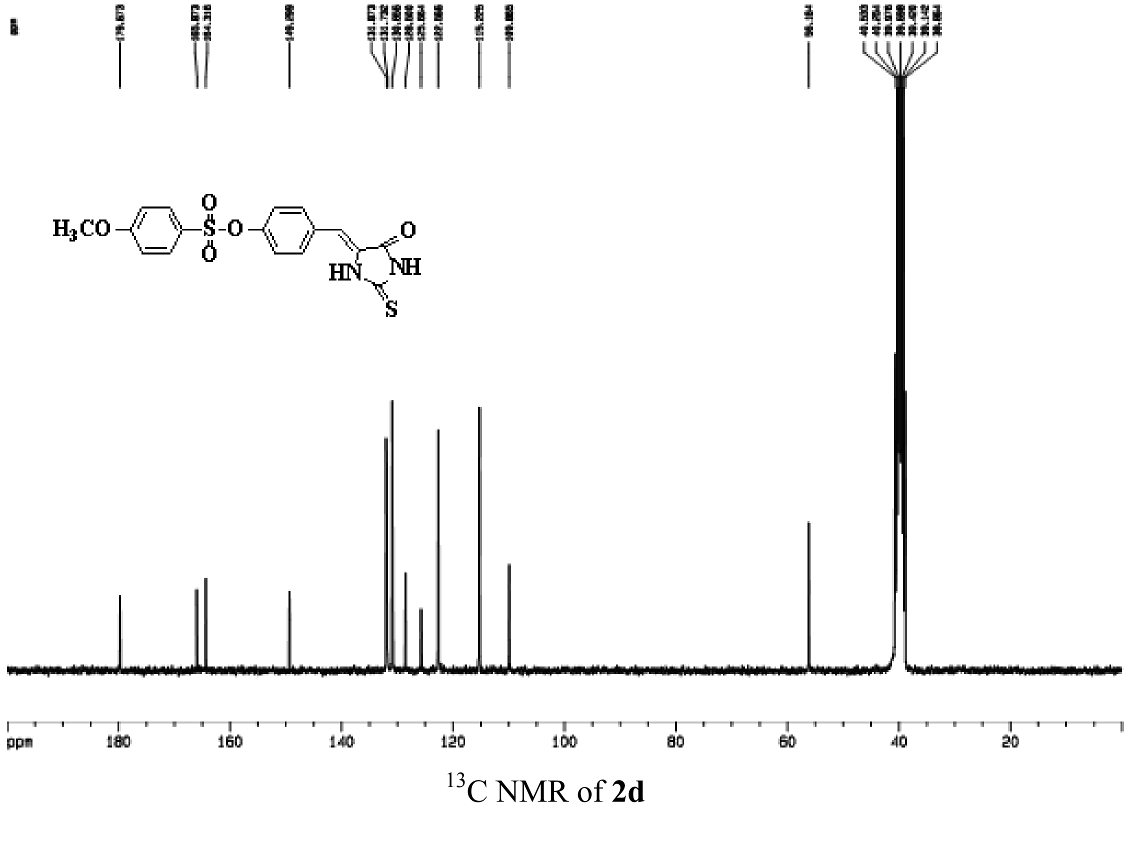

^{13}C NMR of **2d**

Figure S1. *Cont.*

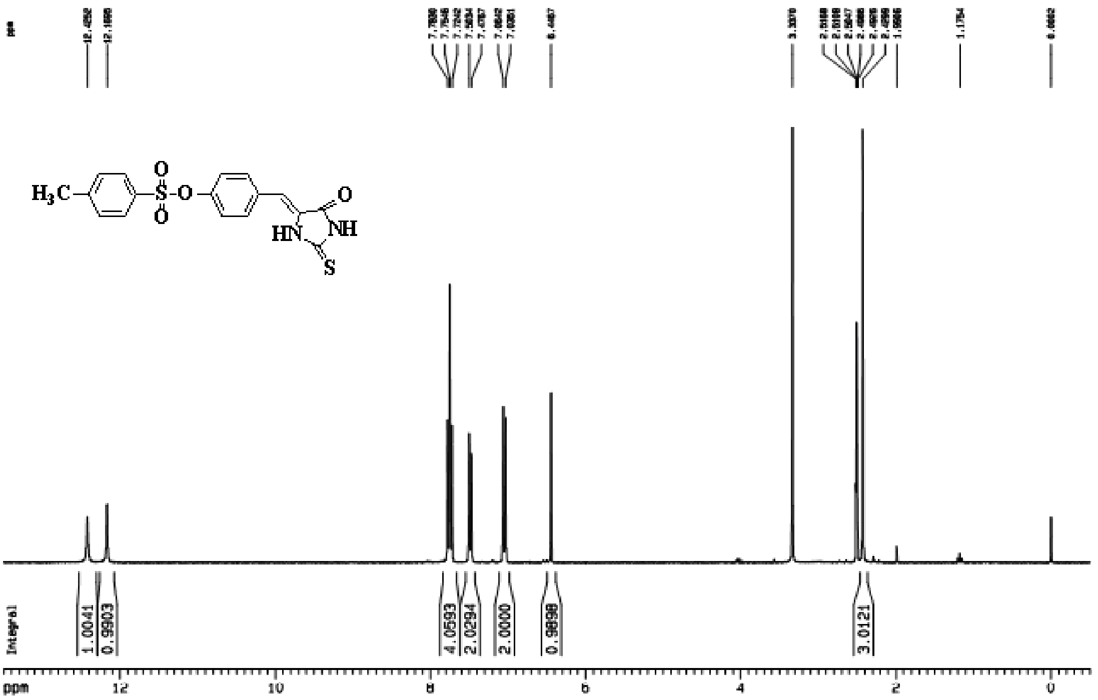

^{1}H NMR of **2e**

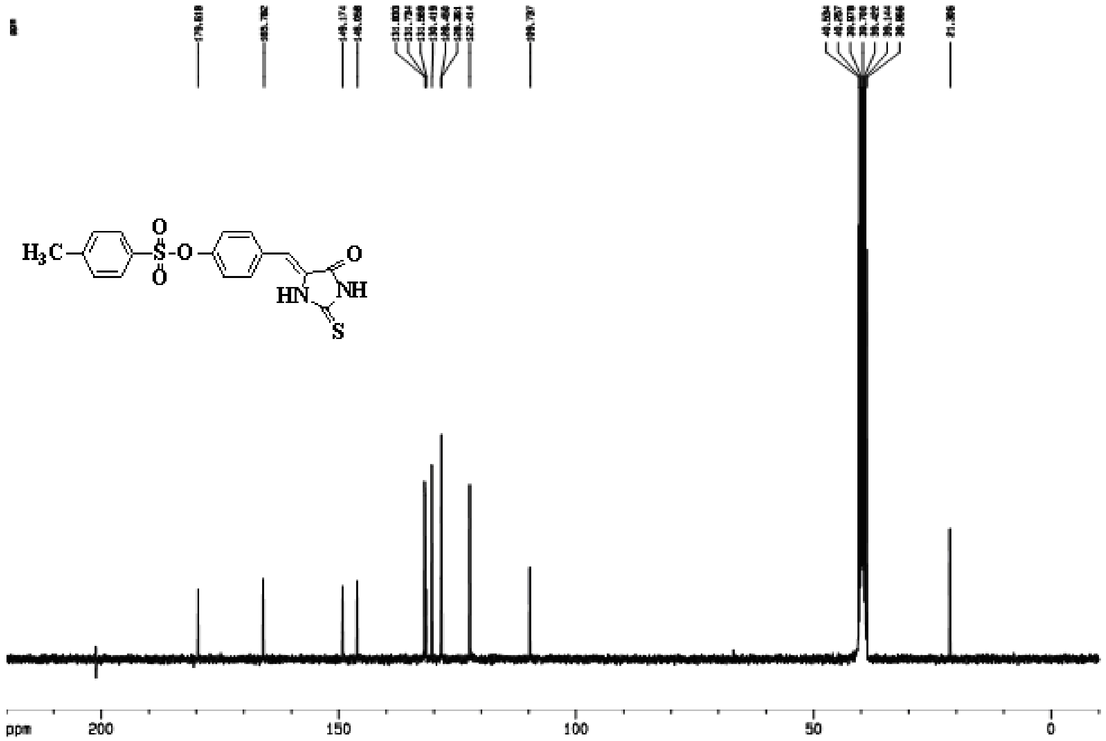

^{13}C NMR of **2e**

Figure S1. *Cont.*

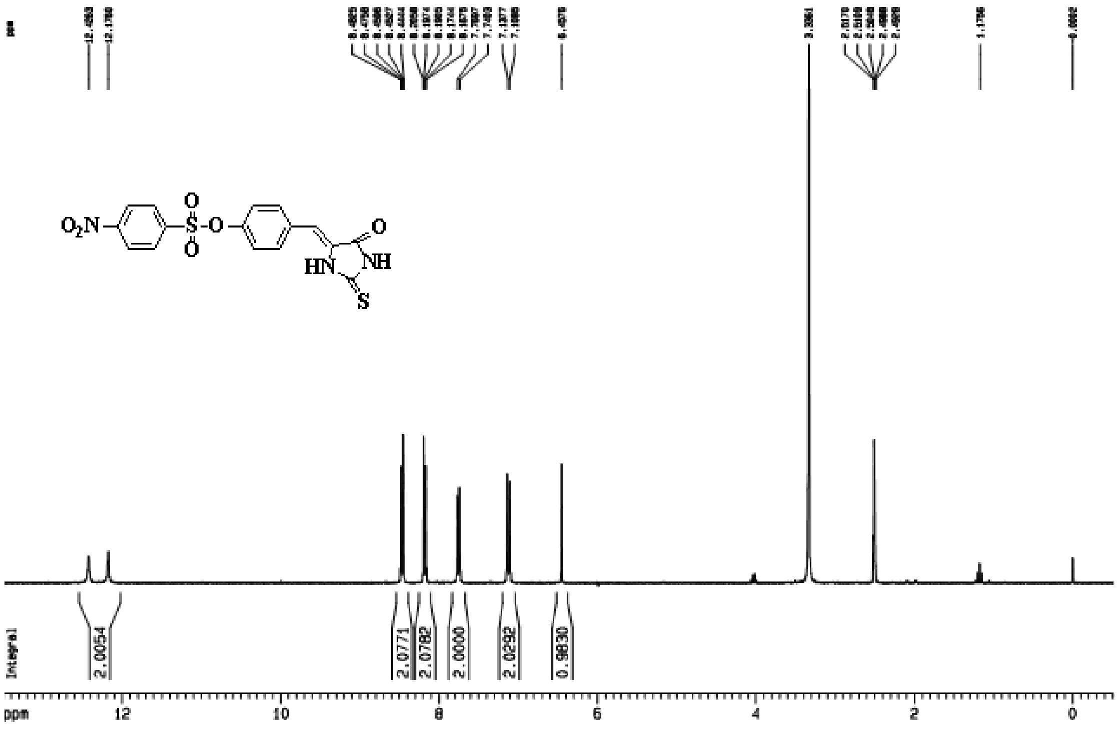

^{1}H NMR of **2f**

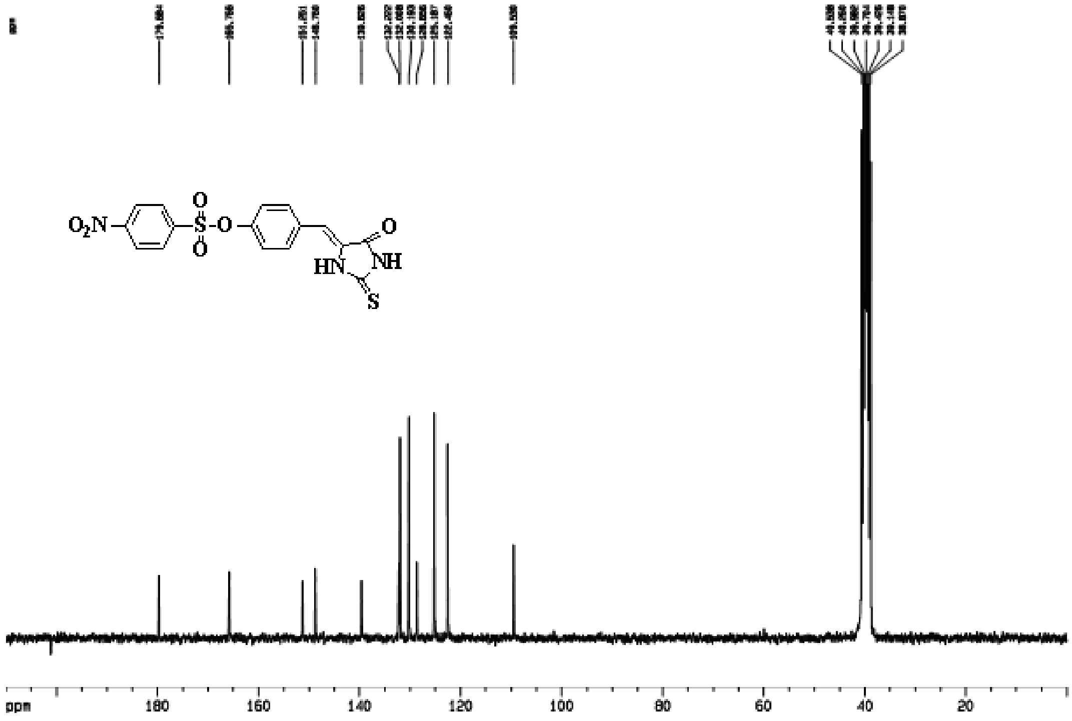

^{13}C NMR of **2f**

Figure S1. *Cont.*

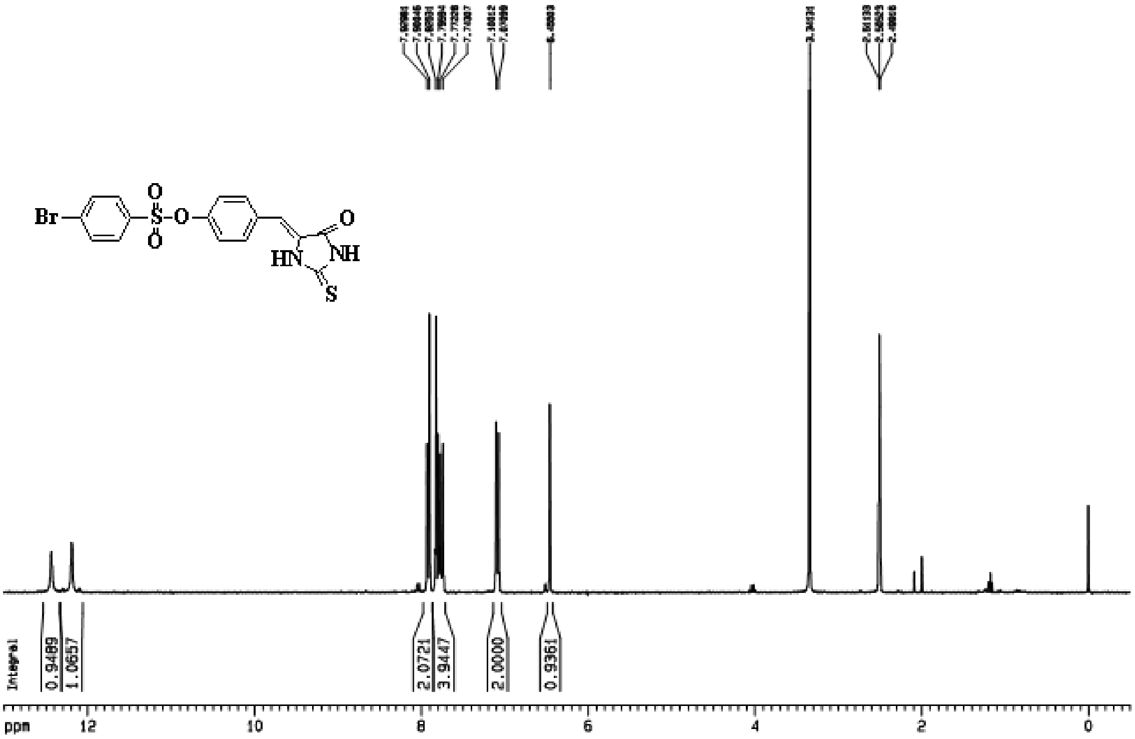

^{1}H NMR of **2g**

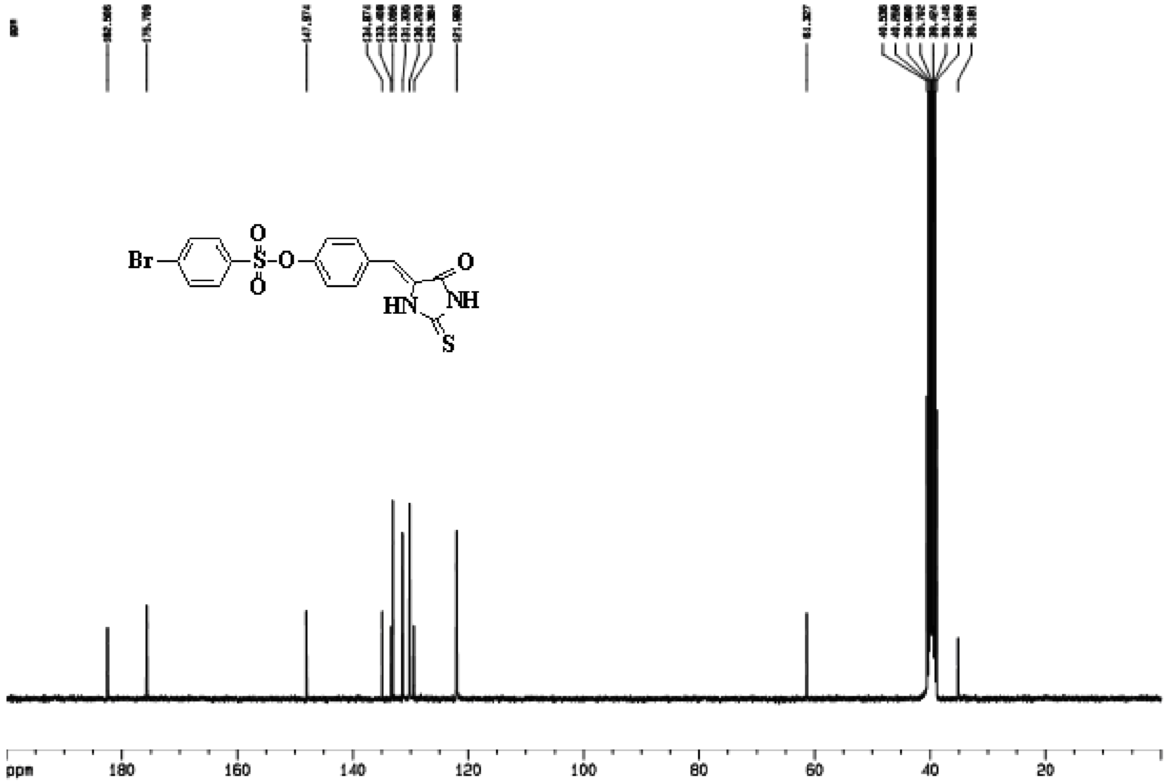

^{13}C NMR of **2g**

Figure S1. *Cont.*

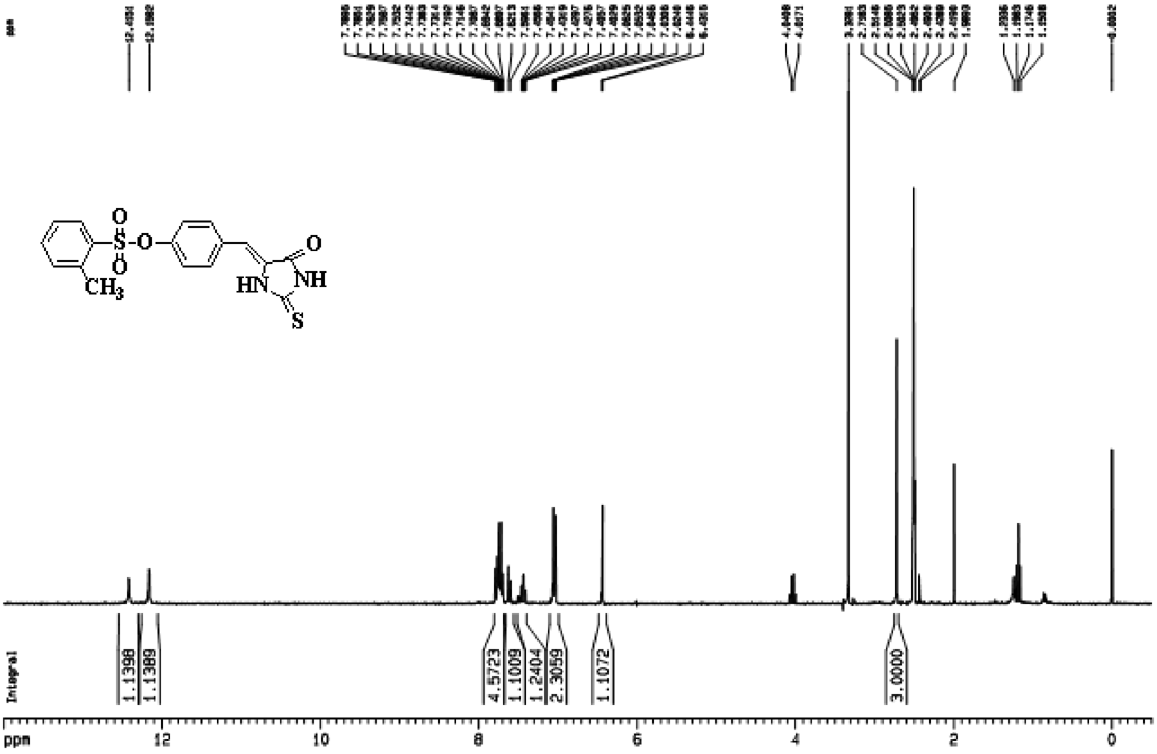

^{1}H NMR of **2h**

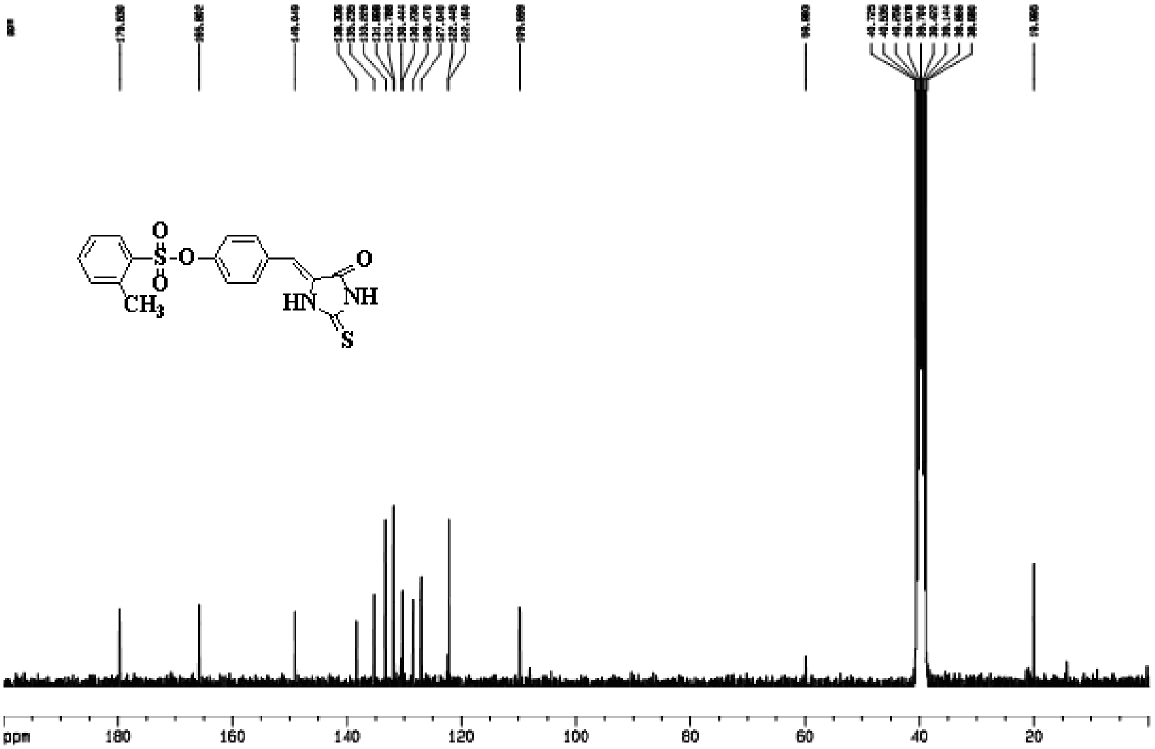

^{13}C NMR of **2h**

Figure S1. *Cont.*

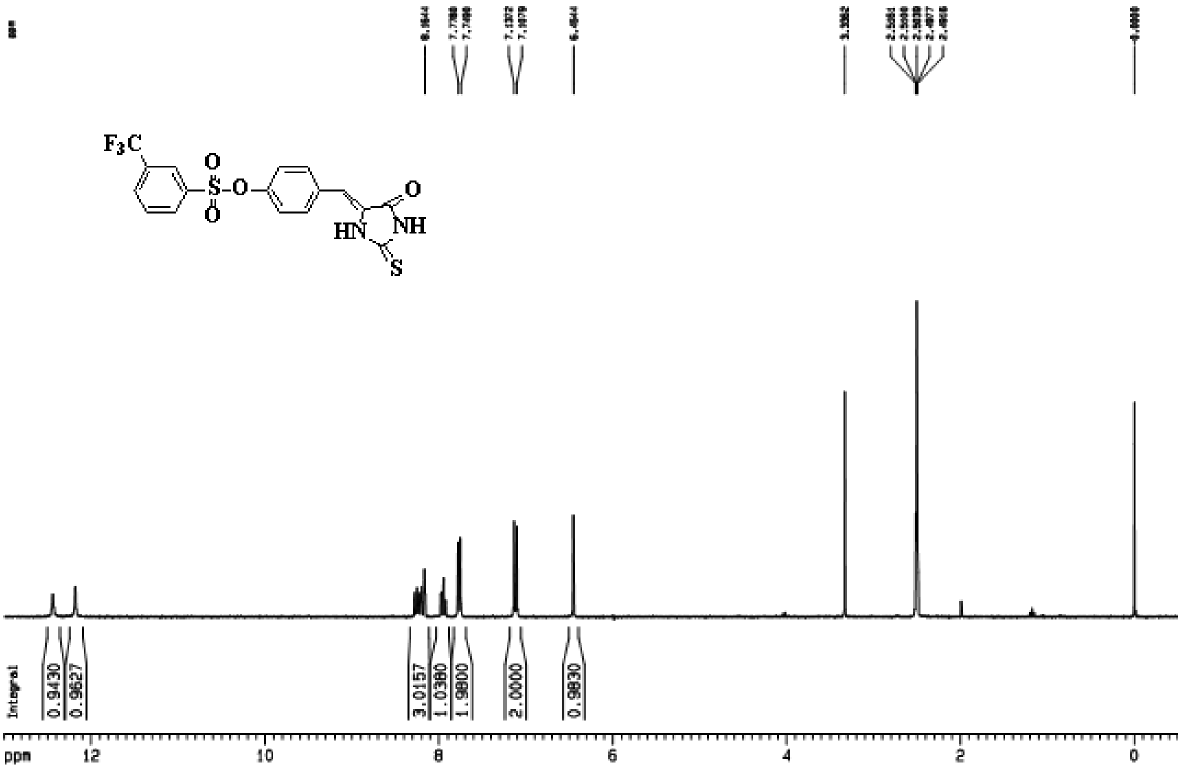

^{1}H NMR of **2i**

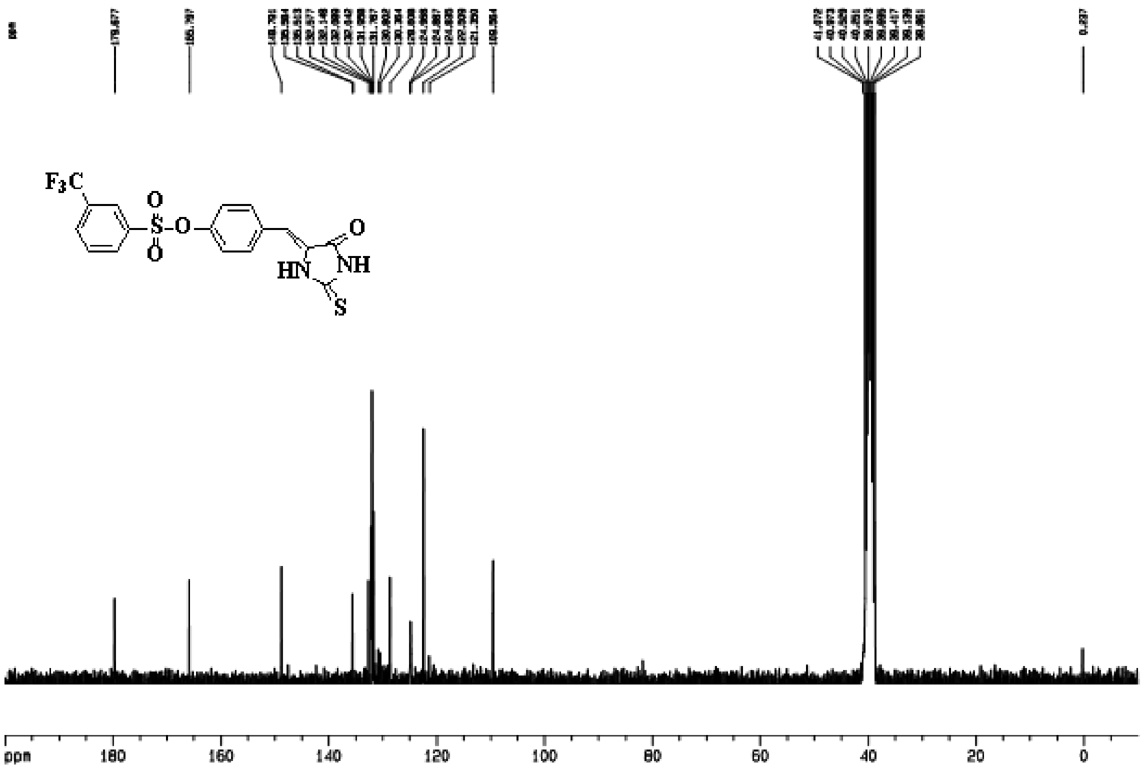

^{13}C NMR of **2i**

Figure S1. *Cont.*

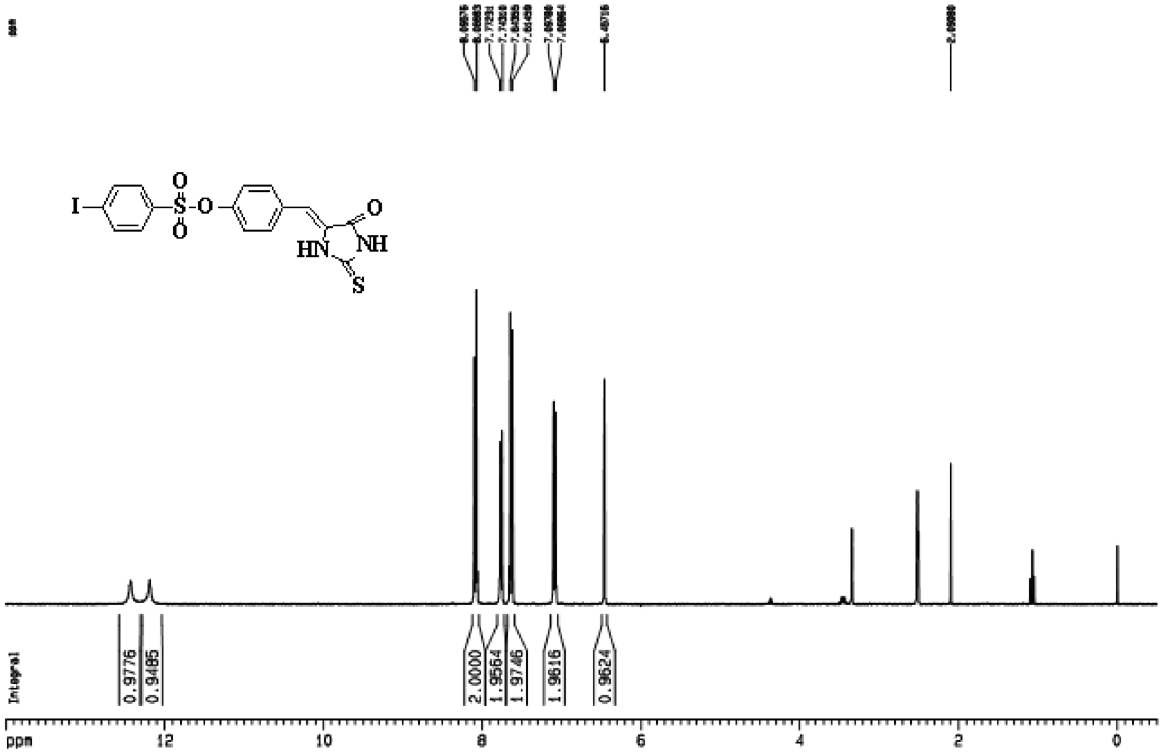

^{1}H NMR of **2j**

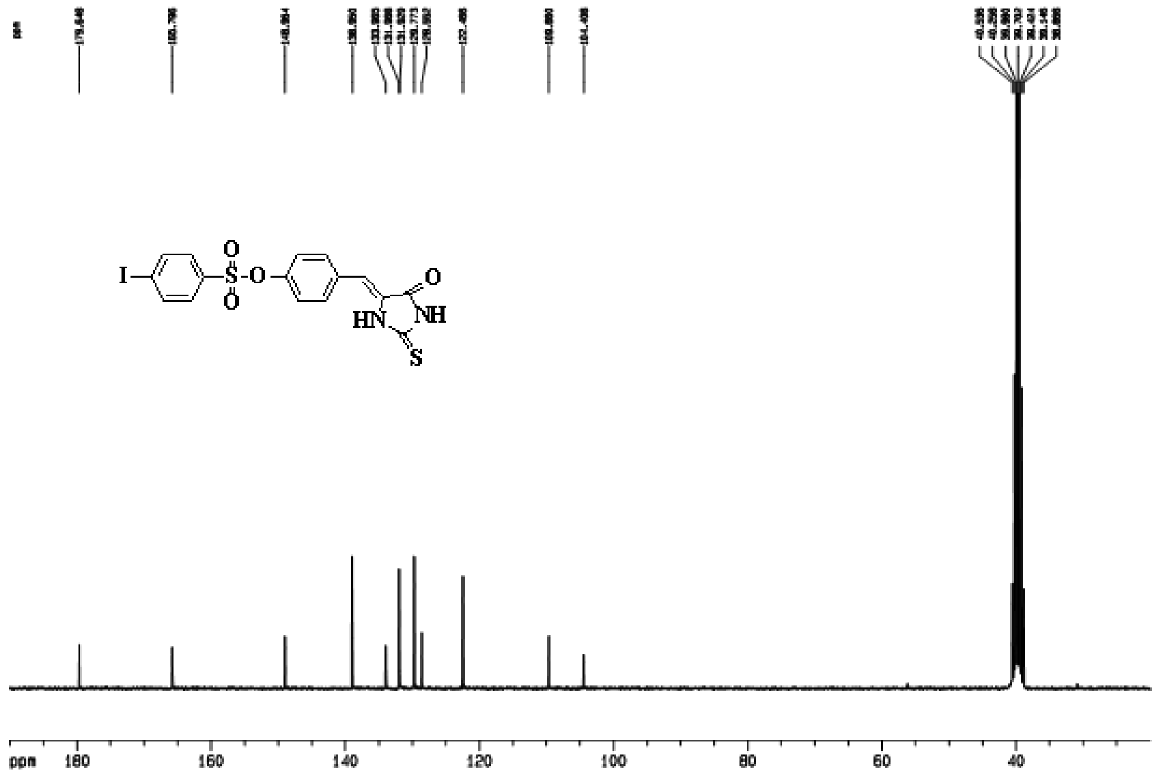

^{13}C NMR of **2j**

Figure S1. *Cont.*

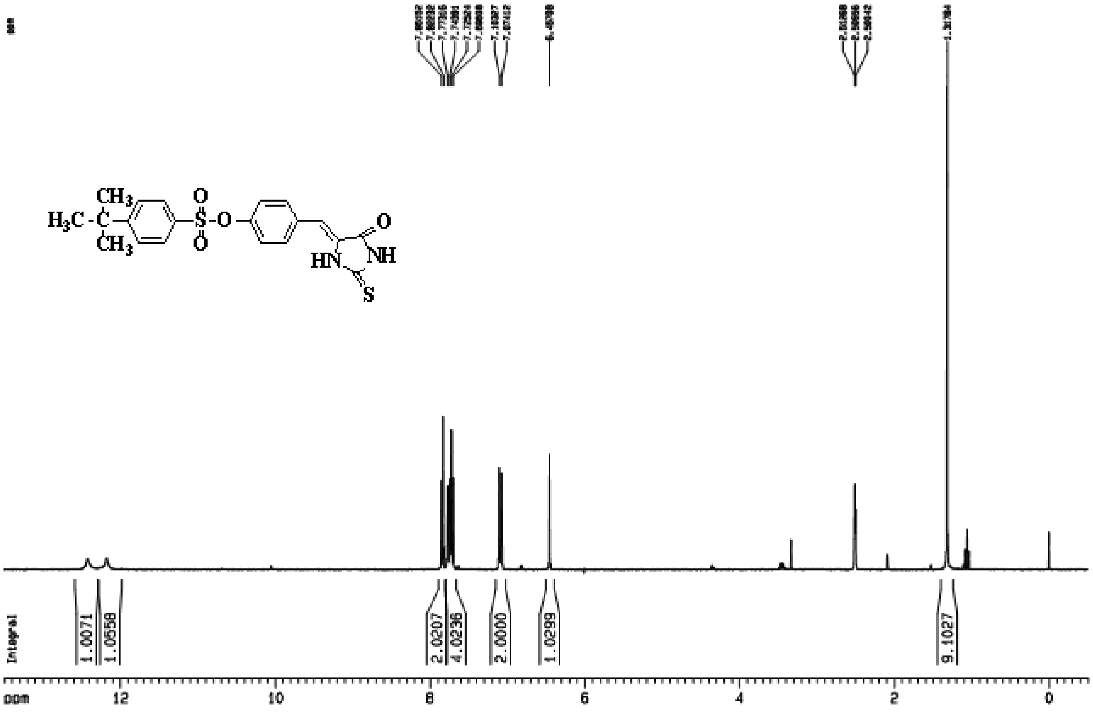

^{1}H NMR of **2k**

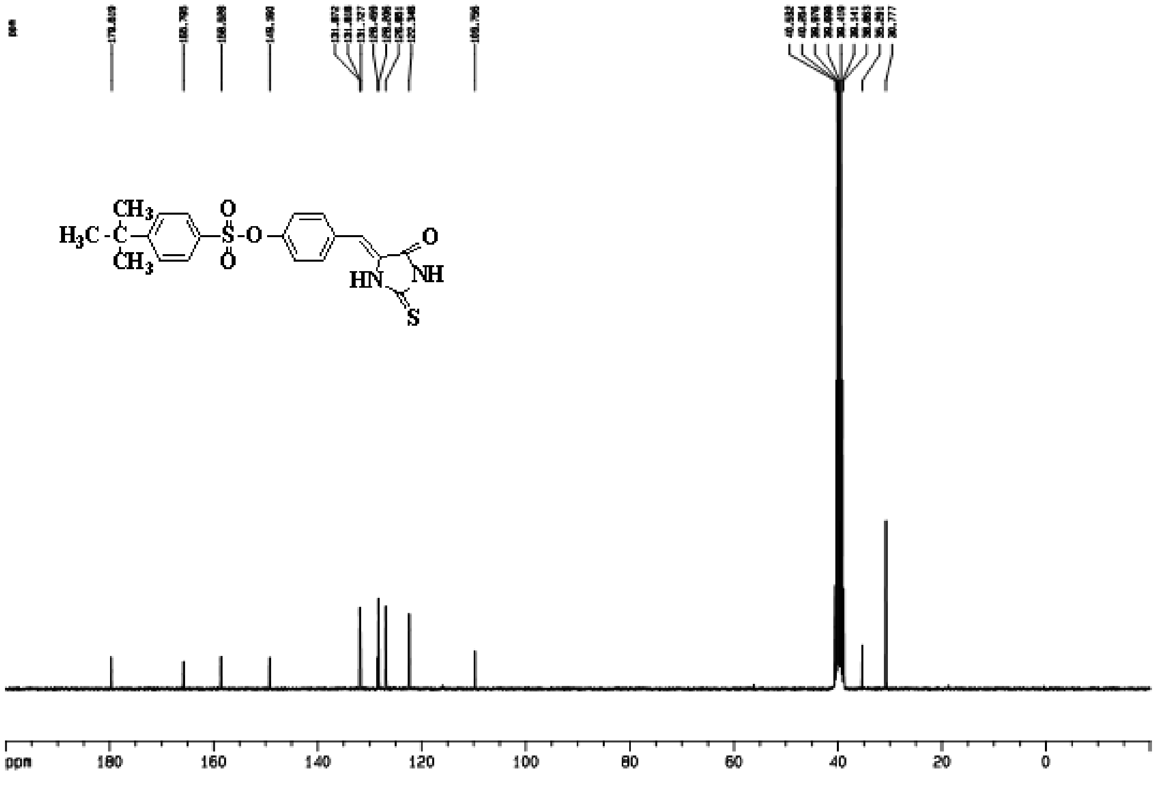

^{13}C NMR of **2k**

Figure S1. *Cont.*

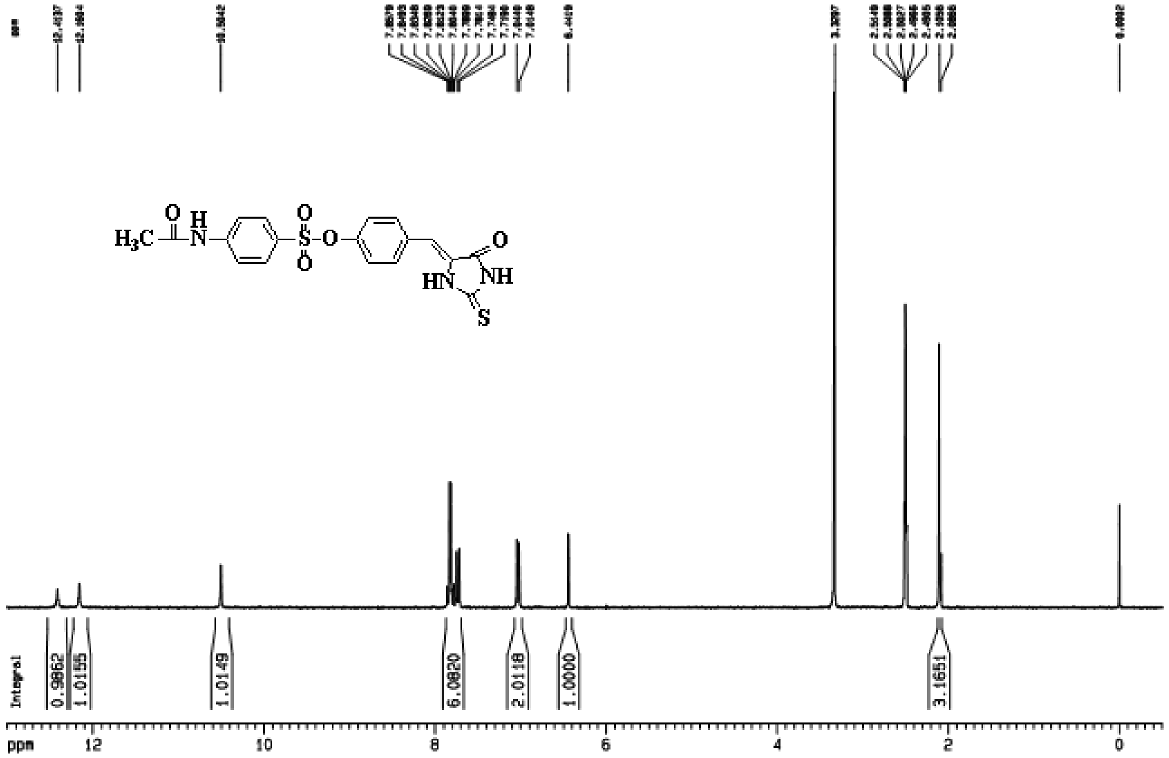

^{1}H NMR of **2l**

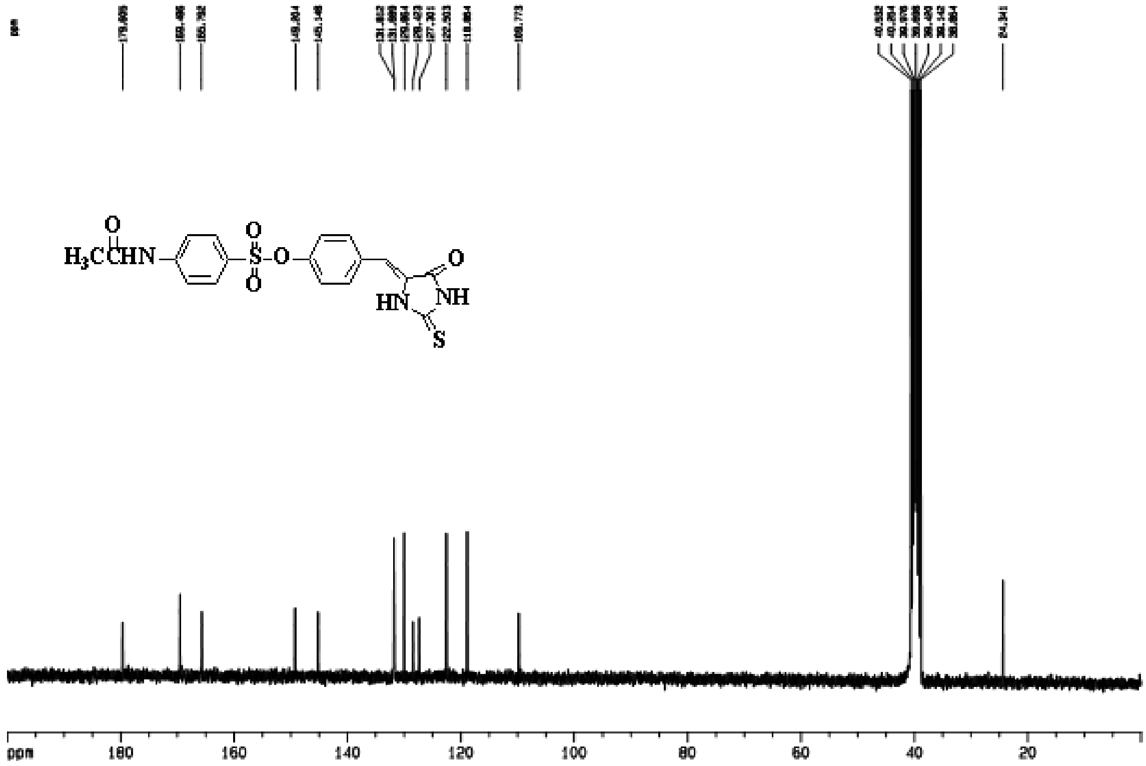

^{13}C NMR of **2l**

Figure S1. *Cont.*

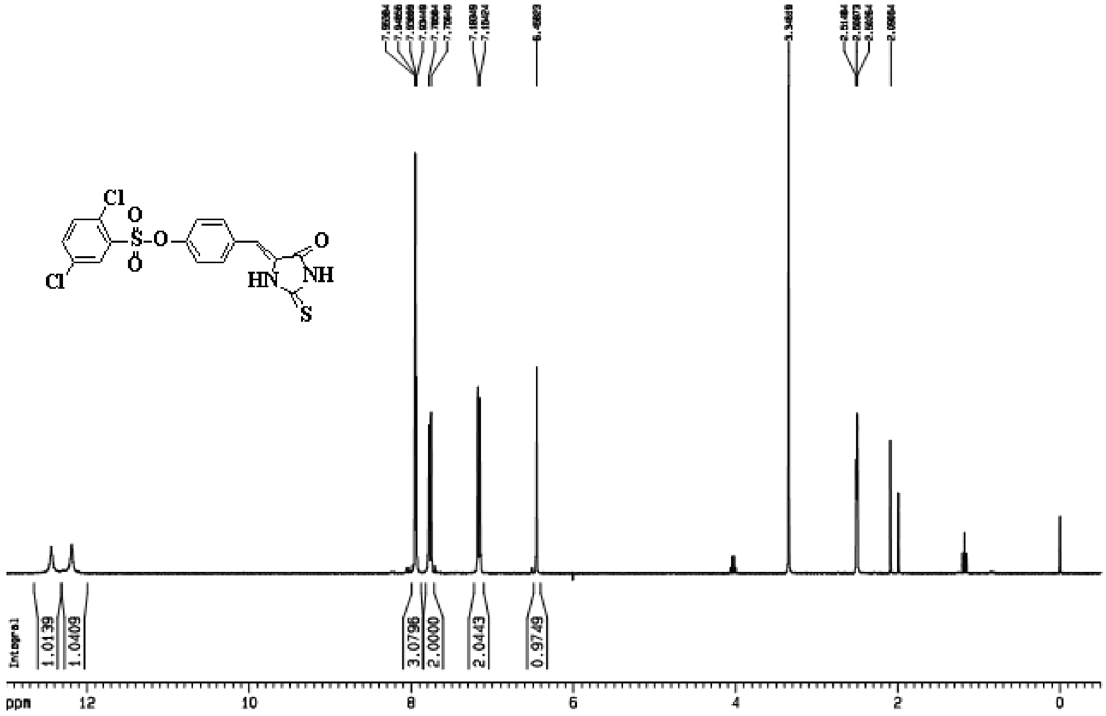

^{1}H NMR of **2m**

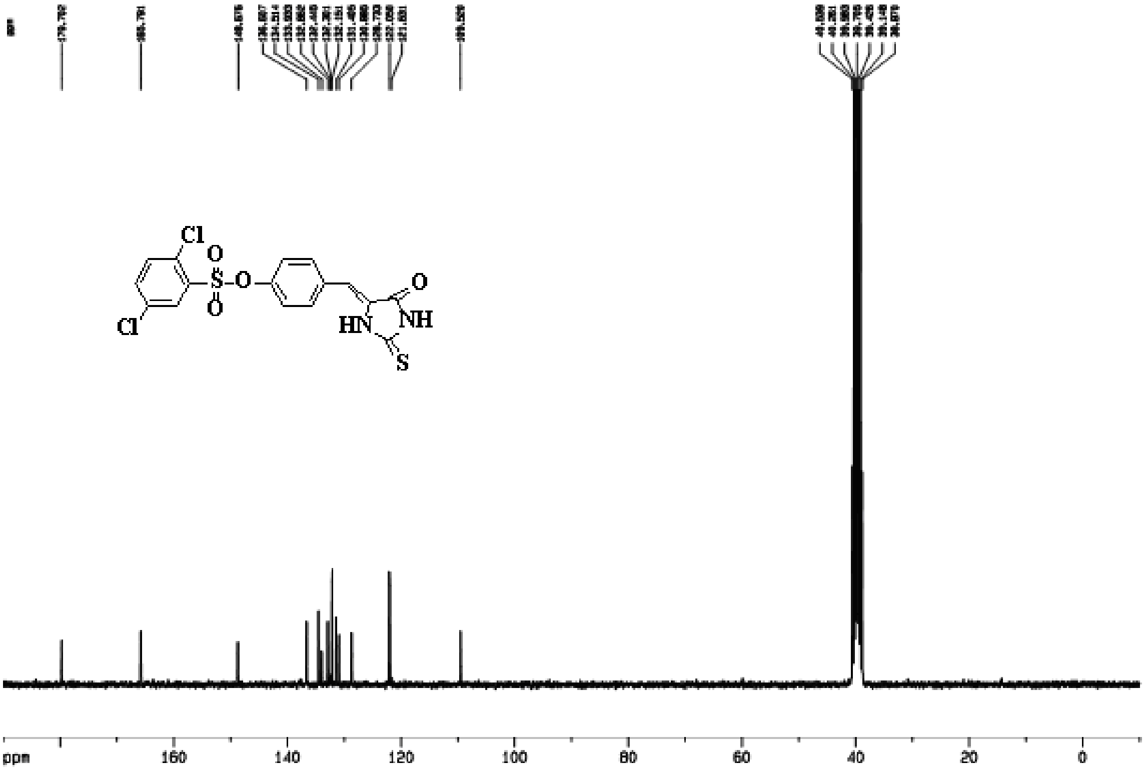

^{13}C NMR of **2m**

Figure S1. *Cont.*

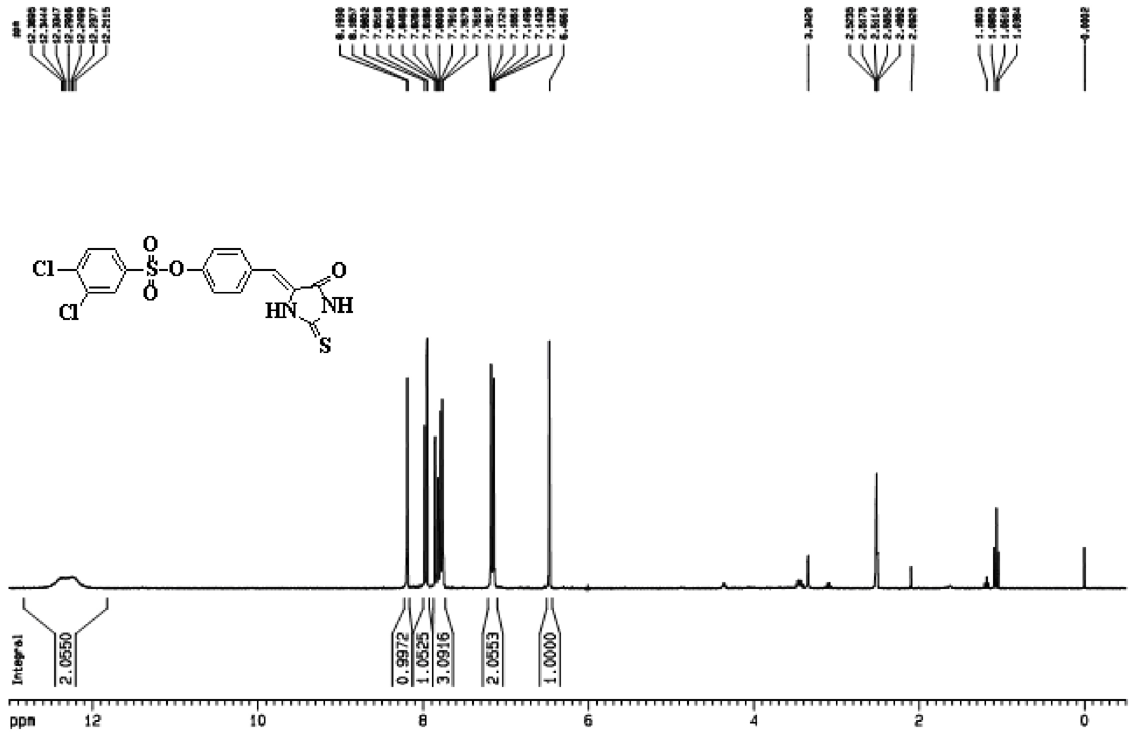

^{1}H NMR of **2n**

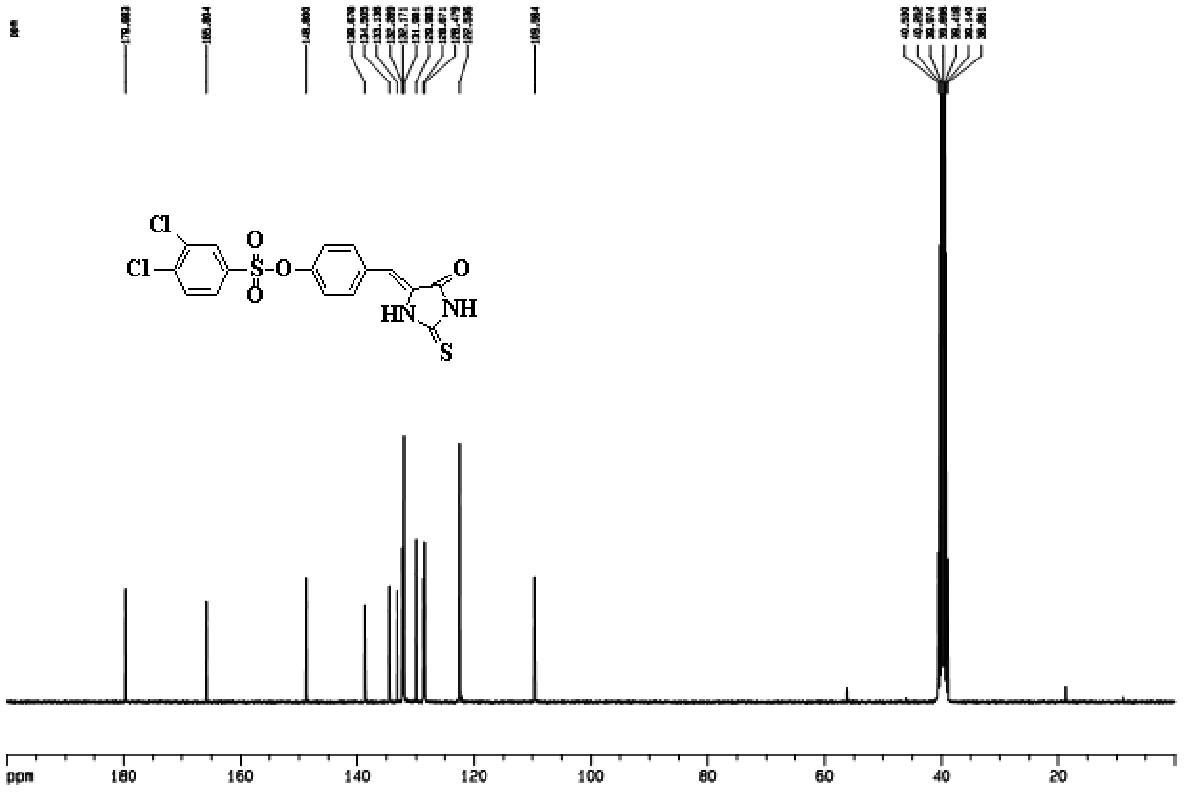

^{13}C NMR of **2n**

Figure S1. *Cont.*

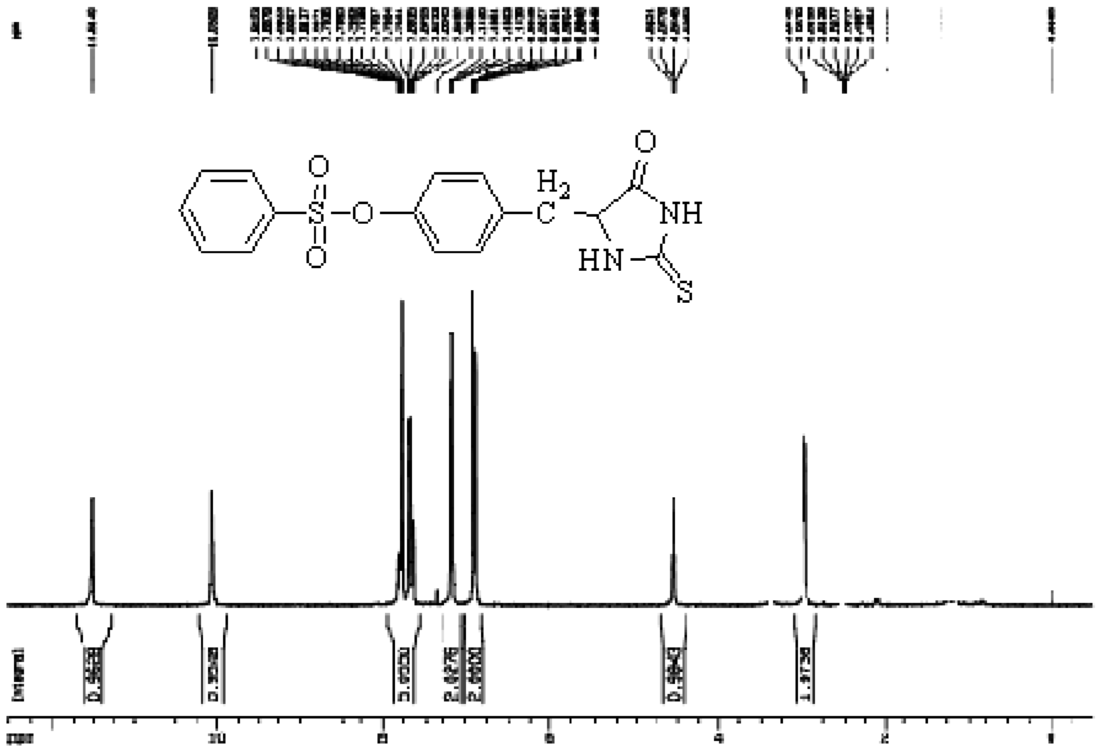

^{1}H NMR of **3a**

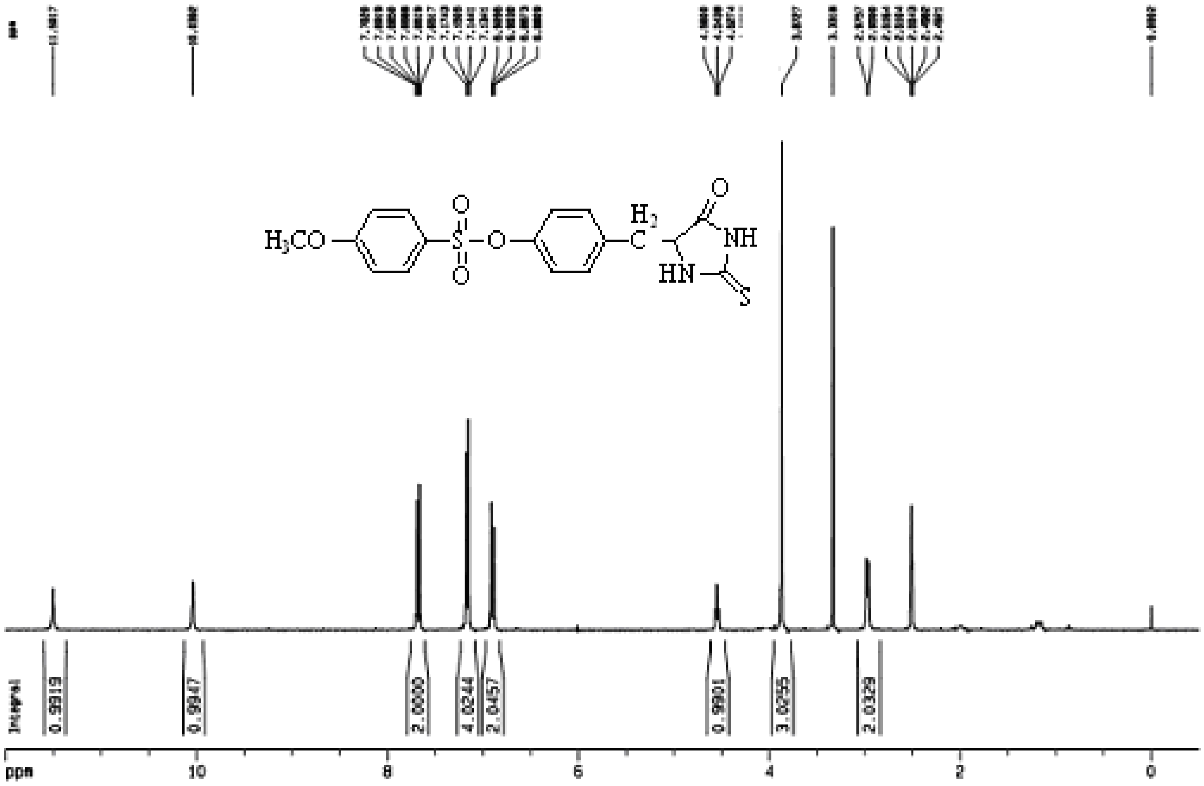

^{1}H NMR of **3d**

Figure S1. *Cont.*

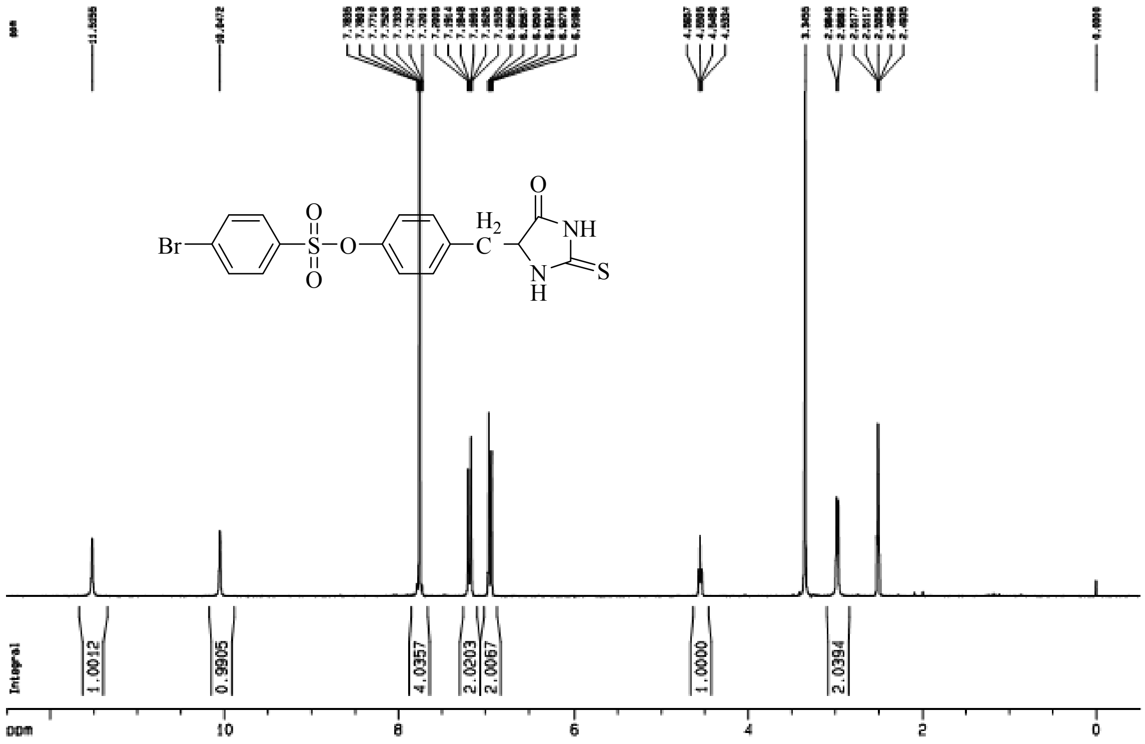

^{1}H NMR of **3g**

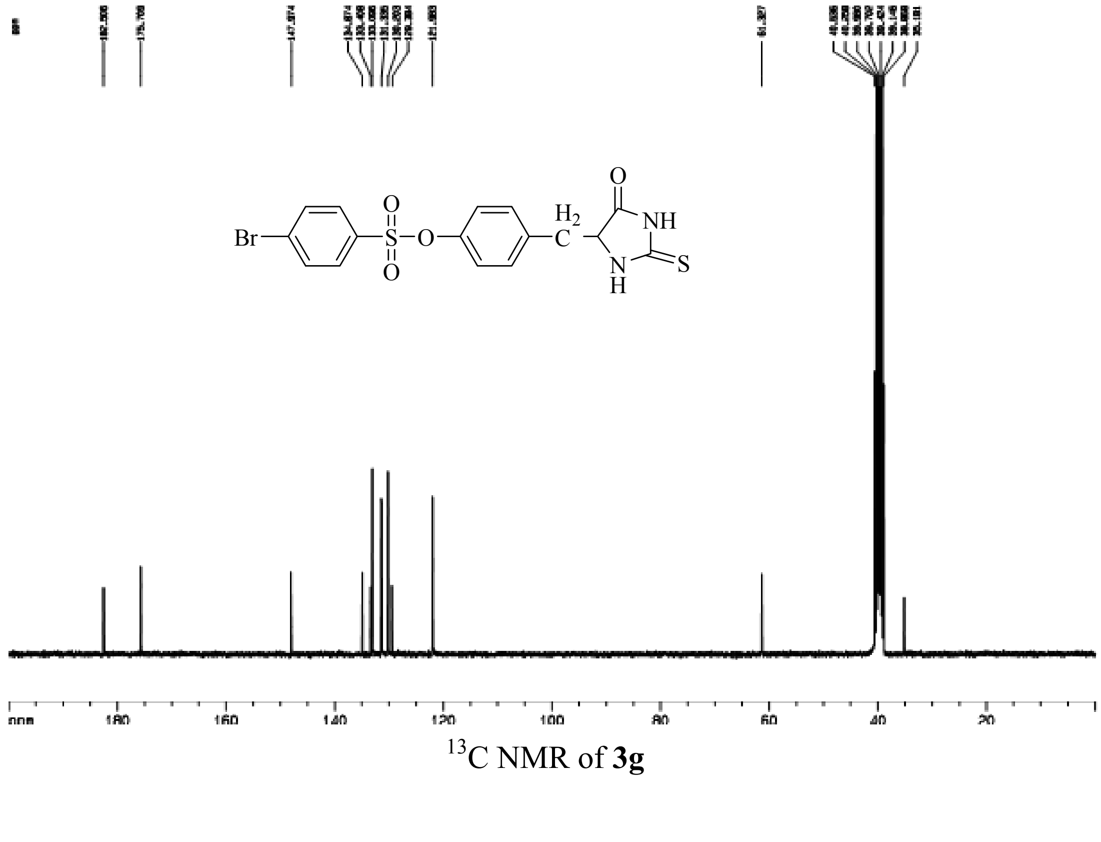

^{13}C NMR of **3g**

Figure S1. *Cont.*

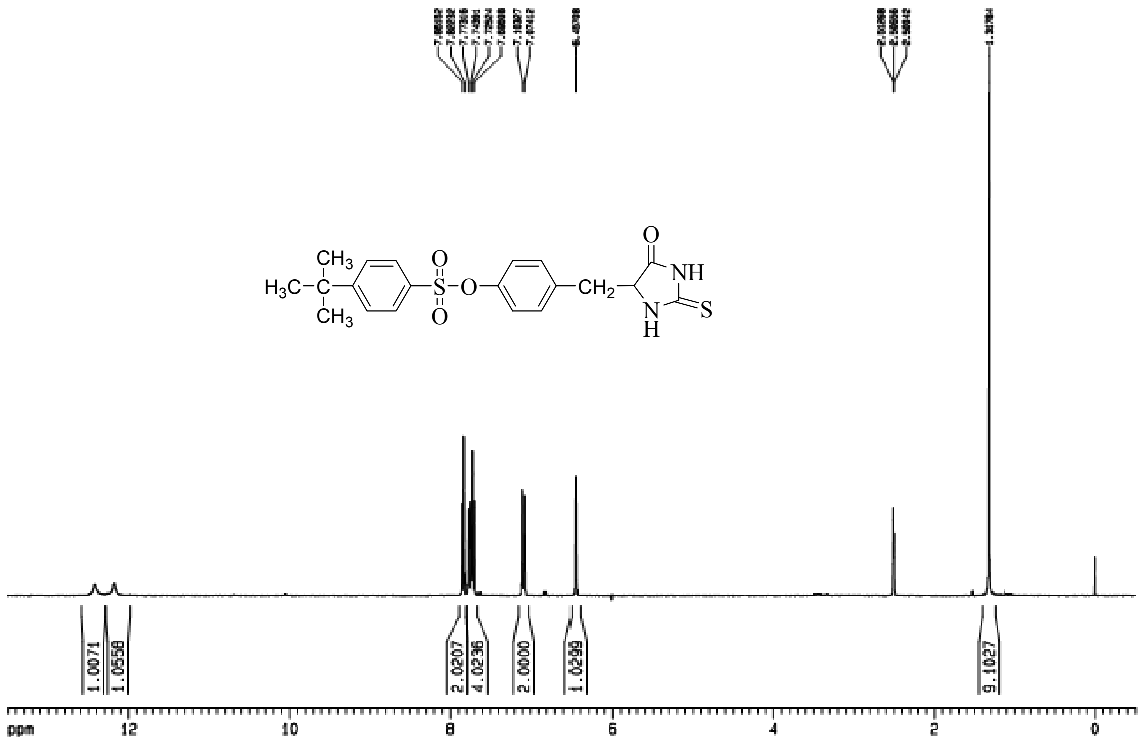

^{1}H NMR of **3k**

Figure S2. X-ray structure and parameters of **2k**. (**a**) X-ray structure of **2k**; (**b**) The packing diagram in the unit cell of **2k**.

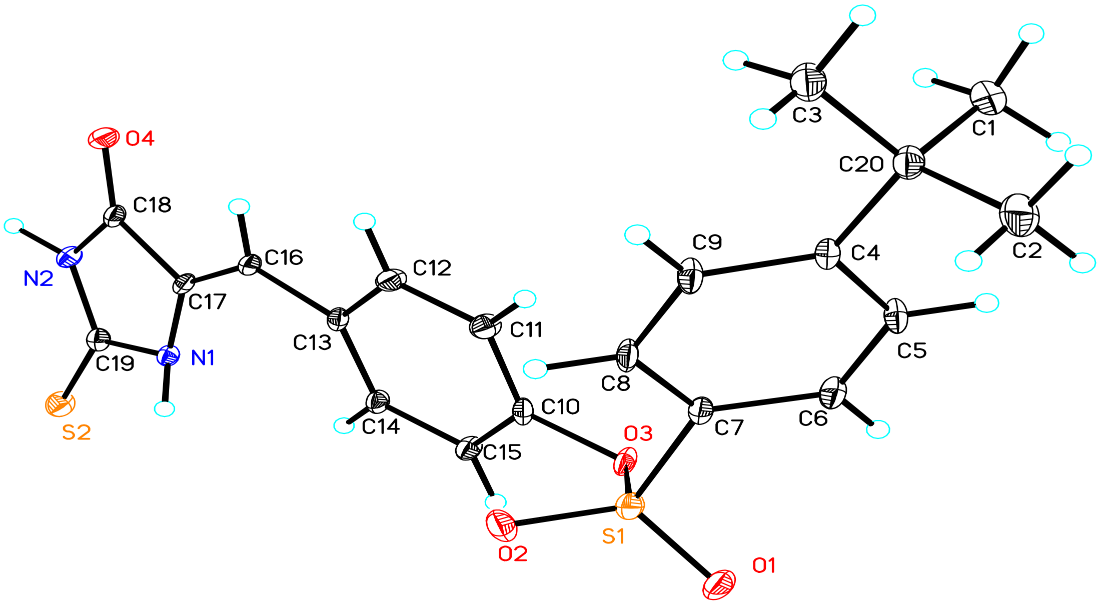

(**a**)

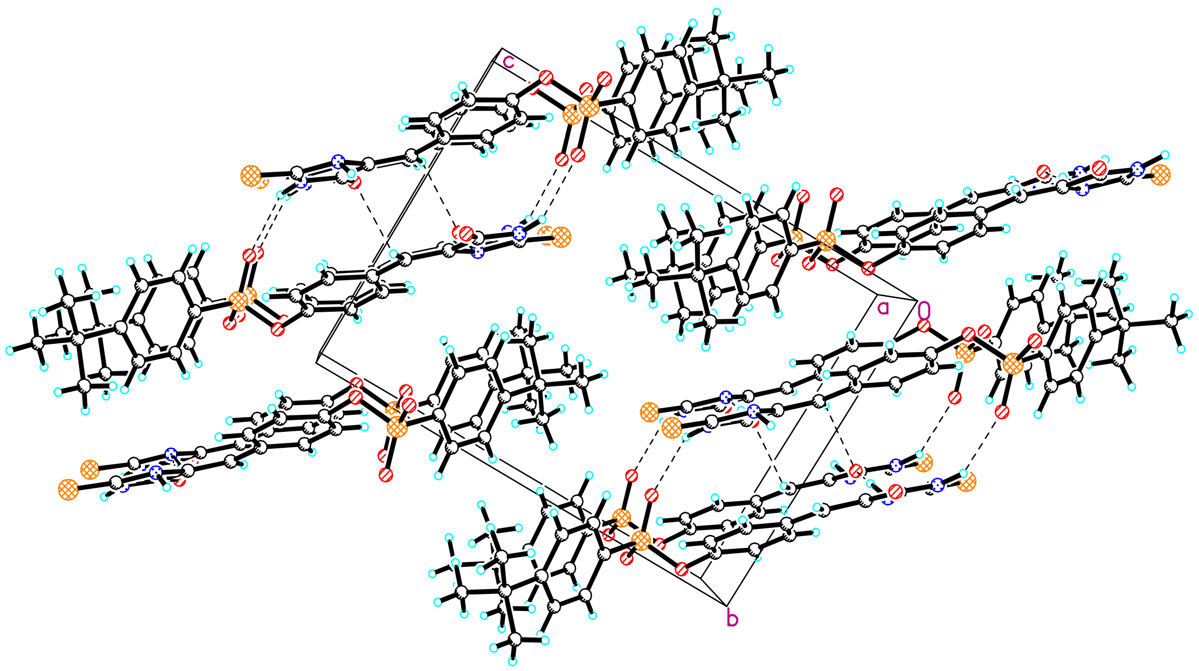

(**b**)

Table S1. Crystal data and structure refinement for **2k**.

Cell parameters	Data
Identification code	**2k**
Empirical formula	C20 H20 N2 O4 S2
Formula weight	416.50
Temperature	293(2) K
Wavelength	0.71073 A
Crystal system, space group	Triclinic, P-1
	a = 6.1500(12) A, alpha = 88.33(3) deg.;
Unit cell dimensions	b = 10.860(2) A, beta = 88.53(3) deg.;
	c = 14.610(3) A, gamma = 81.81(3) deg.
Volume	965.2(3) A^3
Z, Calculated density	2, 1.433mg/m^3
Absorption coefficient	0.306 mm^{-1}
F(000)	436
Crystal size	0.12 × 0.10 × 0.08 mm
Theta range for data collection	1.90 to 24.99 deg.
Limiting indices	$-7 \leq h \leq 7$, $-12 \leq k \leq 9$, $-17 \leq l \leq 15$
Reflections collected/unique	4405/2899 [R(int) = 0.0553]
Completeness to theta = 24.99	85.3%
Absorption correction	None
Max. and min. transmission	0.9760 and 0.9642
Refinement method	Full-matrix least-squares on F^2
Data/restraints/parameters	2899/2/262
Goodness-of-fit on F^2	1.001
Final R indices [I > 2sigma(I)]	*R1* = 0.0590, wR2 = 0.1194
R indices (all data)	*R1* = 0.0943, wR2 = 0.1312
Largest diff. peak and hole	0.349 and −0.303 e.A^{-3}

Table S2. Atomic coordinates (×10^4) and equivalent isotropic displacement parameters (A^2 × 10^3) for **2k**.

	x	*y*	*z*	*U(eq)*
C(1)	4759(8)	13533(5)	3746(3)	79(1)
C(2)	7286(9)	13316(5)	5052(3)	82(2)
C(3)	4666(9)	11786(5)	4852(3)	83(2)
C(4)	7952(7)	11833(4)	3782(2)	48(1)
C(5)	9218(7)	12445(4)	3157(3)	57(1)
C(6)	10866(7)	11810(4)	2618(3)	54(1)
C(7)	11243(6)	10534(4)	2710(2)	44(1)
C(8)	10042(7)	9907(4)	3318(3)	57(1)
C(9)	8405(7)	10565(4)	3850(3)	58(1)
C(10)	11434(7)	8894(4)	699(2)	47(1)
C(11)	9448(6)	8669(4)	1058(2)	51(1)
C(12)	8371(6)	7795(4)	669(2)	48(1)
C(13)	9285(6)	7135(3)	-92(2)	41(1)
C(14)	11297(6)	7382(4)	-442(3)	46(1)
C(15)	12381(6)	8267(4)	-63(2)	46(1)
C(16)	7953(6)	6276(3)	-477(2)	41(1)
C(17)	8397(6)	5457(3)	-1153(2)	39(1)
C(18)	6660(6)	4781(4)	-1489(2)	44(1)
C(19)	9820(6)	4228(4)	-2311(2)	47(1)
C(20)	6167(7)	12602(4)	4363(3)	58(1)
N(1)	10218(4)	5072(3)	-1698(2)	41(1)
N(2)	7660(5)	4085(3)	-2181(2)	49(1)
O(1)	15139(5)	10459(3)	1997(2)	72(1)
O(2)	13780(5)	8470(3)	2345(2)	65(1)
O(3)	12550(4)	9825(2)	1038(2)	52(1)
O(4)	4722(4)	4852(3)	-1227(2)	60(1)
S(1)	13396(2)	9739(1)	2052(1)	53(1)
S(2)	11564(2)	3526(1)	-3056(1)	64(1)

U(eq) is defined as one third of the trace of the orthogonalized Uij tensor.

Table S3. Bond lengths [A] and angles [deg] for **2k**.

**Bond or angle**		**Bond or angle**		**Bond or angle**	
C(1)–C(20)	1.524(6)	C(6)–C(7)	1.376(5)	C(14)–H(14)	0.9300
C(1)–H(1A)	0.9600	C(6)–H(6)	0.9300	C(15)–H(15)	0.9300
C(1)–H(1B)	0.9600	C(7)–C(8)	1.368(5)	C(16)–C(17)	1.346(5)
C(1)–H(1C)	0.9600	C(7)–S(1)	1.755(4)	C(16)–H(16)	0.9300
C(2)–C(20)	1.524(6)	C(8)–C(9)	1.384(6)	C(17)–N(1)	1.380(4)
C(2)–H(2A)	0.9600	C(8)–H(8)	0.9300	C(17)–C(18)	1.481(5)
C(2)–H(2B)	0.9600	C(9)–H(9)	0.9300	C(18)–O(4)	1.236(4)
C(2)–H(2C)	0.9600	C(10)–C(11)	1.368(5)	C(18)–N(2)	1.361(5)
C(3)–C(20)	1.520(6)	C(10)–C(15)	1.393(5)	C(19)–N(1)	1.352(5)
C(3)–H(3A)	0.9600	C(10)–O(3)	1.408(4)	C(19)–N(2)	1.367(5)
C(3)–H(3B)	0.9600	C(11)–C(12)	1.376(5)	C(19)–S(2)	1.634(4)
C(3)–H(3C)	0.9600	C(11)–H(11)	0.9300	N(1)–H(1N)	0.9700(4)
C(4)–C(9)	1.367(5)	C(12)–C(13)	1.402(5)	N(2)–H(2N)	0.9701(6)
C(4)–C(5)	1.399(6)	C(12)–H(12)	0.9300	O(1)–S(1)	1.413(3)
C(4)–C(20)	1.534(5)	C(13)–C(14)	1.385(5)	O(2)–S(1)	1.420(3)
C(5)–C(6)	1.384(5)	C(13)–C(16)	1.459(5)	O(3)–S(1)	1.580(3)
C(5)–H(5)	0.9300	C(14)–C(15)	1.382(5)		
C(20)–C(1)–H(1A)	109.5	C(6)–C(7)–S(1)	117.6(3)	C(16)–C(17)–C(18)	120.5(3)
C(20)–C(1)–H(1B)	109.5	C(7)–C(8)–C(9)	119.6(4)	N(1)–C(17)–C(18)	104.8(3)
H(1A)–C(1)–H(1B)	109.5	C(7)–C(8)–H(8)	120.2	O(4)–C(18)–N(2)	127.3(3)
C(20)–C(1)–H(1C)	109.5	C(9)–C(8)–H(8)	120.2	O(4)–C(18)–C(17)	128.4(3)
H(1A)–C(1)–H(1C)	109.5	C(4)–C(9)–C(8)	121.4(4)	N(2)–C(18)–C(17)	104.3(3)
H(1B)–C(1)–H(1C)	109.5	C(4)–C(9)–H(9)	119.3	N(1)–C(19)–N(2)	105.8(3)
C(20)–C(2)–H(2A)	109.5	C(8)–C(9)–H(9)	119.3	N(1)–C(19)–S(2)	127.0(3)
C(20)–C(2)–H(2B)	109.5	C(11)–C(10)–C(15)	121.3(4)	N(2)–C(19)–S(2)	127.2(3)
H(2A)–C(2)–H(2B)	109.5	C(11)–C(10)–O(3)	122.0(3)	C(3)–C(20)–C(2)	110.5(4)
C(20)–C(2)–H(2C)	109.5	C(15)–C(10)–O(3)	116.6(4)	C(3)–C(20)–C(1)	107.9(4)
H(2A)–C(2)–H(2C)	109.5	C(10)–C(11)–C(12)	119.6(4)	C(2)–C(20)–C(1)	108.7(4)
H(2B)–C(2)–H(2C)	109.5	C(10)–C(11)–H(11)	120.2	C(3)–C(20)–C(4)	111.7(4)
C(20)–C(3)–H(3A)	109.5	C(12)–C(11)–H(11)	120.2	C(2)–C(20)–C(4)	108.4(4)
C(20)–C(3)–H(3B)	109.5	C(11)–C(12)–C(13)	120.7(4)	C(1)–C(20)–C(4)	109.5(3)
H(3A)–C(3)–H(3B)	109.5	C(11)–C(12)–H(12)	119.6	C(19)–N(1)–C(17)	112.1(3)
C(20)–C(3)–H(3C)	109.5	C(13)–C(12)–H(12)	119.6	C(19)–N(1)–H(1N)	122(3)
H(3A)–C(3)–H(3C)	109.5	C(14)–C(13)–C(12)	118.5(3)	C(17)–N(1)–H(1N)	111(2)
H(3B)–C(3)–H(3C)	109.5	C(14)–C(13)–C(16)	125.4(3)	C(18)–N(2)–C(19)	112.9(3)
C(9)–C(4)–C(5)	117.5(4)	C(12)–C(13)–C(16)	116.1(3)	C(18)–N(2)–H(2N)	115(2)
C(9)–C(4)–C(20)	123.3(4)	C(15)–C(14)–C(13)	121.3(3)	C(19)–N(2)–H(2N)	130(2)
C(5)–C(4)–C(20)	119.2(4)	C(15)–C(14)–H(14)	119.4	C(10)–O(3)–S(1)	120.8(3)
C(6)–C(5)–C(4)	122.3(4)	C(13)–C(14)–H(14)	119.4	O(2)–S(1)–O(1)	120.52(19)
C(6)–C(5)–H(5)	118.9	C(14)–C(15)–C(10)	118.6(4)	O(2)–S(1)–O(3)	109.09(15)
C(4)–C(5)–H(5)	118.9	C(14)–C(15)–H(15)	120.7	O(1)–S(1)–O(3)	102.50(18)
C(7)–C(6)–C(5)	117.9(4)	C(10)–C(15)–H(15)	120.7	O(2)–S(1)–C(7)	109.69(19)
C(7)–C(6)–H(6)	121.1	C(17)–C(16)–C(13)	130.9(3)	O(1)–S(1)–C(7)	108.52(18)
C(5)–C(6)–H(6)	121.1	C(17)–C(16)–H(16)	114.5	O(3)–S(1)–C(7)	105.35(16)
C(6)–C(5)–C(4)	122.3(4)	C(13)–C(14)–H(14)	119.4	O(2)–S(1)–O(1)	120.52(19)
C(8)–C(7)–C(6)	121.3(4)	C(13)–C(16)–H(16)	114.5		
C(8)–C(7)–S(1)	121.0(3)	C(16)–C(17)–N(1)	134.7(3)		

© 2013 by the authors; licensee MDPI, Basel, Switzerland. This article is an open access article distributed under the terms and conditions of the Creative Commons Attribution license (http://creativecommons.org/licenses/by/3.0/).
